# Investigation of the cause of geographic disparities in IDEXX ELISA sensitivity in serum samples from *Mycobacterium bovis*-infected cattle

**DOI:** 10.1038/srep22763

**Published:** 2016-03-07

**Authors:** Brett Trost, Tod Stuber, Om Surujballi, Jeffrey Nelson, Suelee Robbe-Austerman, Noel H. Smith, Louis Desautels, Suresh K. Tikoo, Philip Griebel

**Affiliations:** 1 Vaccine and Infectious Disease Organization, University of Saskatchewan, Saskatoon, Saskatchewan S7N 5E3, Canada; 2 Department of Computer Science, University of Saskatchewan, Saskatoon, Saskatchewan, Canada; 3 National Veterinary Services Laboratories, Animal and Plant Health Inspection Service, United States Department of Agriculture, Ames, Iowa 50010, USA; 4 Canadian Food Inspection Agency, Ottawa, Ontario K2H 8P9, Canada; 5 Animal and Plant Health Agency, New Haw, Surrey KT15 3NB, United Kingdom; 6 Canadian Cattlemen’s Association, Calgary, Alberta T2E 7H7, Canada; 7 Vaccinology & Immunotherapeutics Program, School of Public Health, University of Saskatchewan, Saskatoon, Saskatchewan S7N 5E3, Canada

## Abstract

Accurately identifying *Mycobacterium bovis*-infected cattle is critical for bovine tuberculosis prevention and control. One method for identifying infected cattle is an ELISA developed by IDEXX laboratories, which detects antibodies to two *M. bovis* proteins, MPB70 and MPB83. The assay’s sensitivity varies by geographic region, with sensitivities of 77%, 45%, and 9% in bovine serum samples from the United Kingdom (*n* = 126), the United States (*n* = 146), and Mexico (*n* = 128), respectively. We hypothesized that geographically-biased sequence variation in *mpb70* and *mpb83*, or in the genes that regulate their expression (*sigK* and *rskA*), may explain these differing sensitivities. This hypothesis was tested by comparing the sequences of these four genes in 455 *M. bovis* strains isolated from cattle in the aforementioned countries. For each gene, a single, common sequence was identified in most genomes of the *M. bovi*s strains collected in all three countries. Twelve of the 455 strains were isolated from infected cattle for which the IDEXX ELISA was also performed. Five of the seven ELISA-positive genomes and three of the five ELISA-negative genomes contained the most common sequence of all four genes. Thus, sequence variation in *mpb70*, *mpb83*, *sigK*, and *rskA* does not explain the geographic disparities in IDEXX ELISA sensitivity.

The bacterium *Mycobacterium bovis* is the etiological agent of bovine tuberculosis, a persistent infection that primarily affects the lungs but can also spread to other body systems. It is a member of the *Mycobacterium tuberculosis* complex, which is a group of bacterial species that cause tuberculosis. As it has a significant impact on both animal health and international trade, substantial economic resources are devoted to preventing, managing, and controlling bovine tuberculosis outbreaks^1^,^2^. In addition to cattle, *M. bovis* can infect many other hosts, including humans^1^,^3^, although *Mycobacterium tuberculosis*, another member of the *Mycobacterium tuberculosis* complex, is responsible for the majority of human tuberculosis cases. Human-to-human transmission of *M. bovis* is rare, with most human infections resulting from the consumption of unpasteurized dairy products or the inhalation of respiratory droplets from infected cattle^4^.

Due to the economic consequences associated with *M. bovis* infections in cattle, as well as its zoonotic potential, methods for quickly and accurately identifying infected cattle are of considerable importance. One such method is an enzyme-linked immunosorbent assay (ELISA) developed by IDEXX Laboratories (Westbrook, ME), which allows for the rapid detection of *M. bovis*-specific antibodies in bovine serum or milk samples^5^,^6^. This assay detects antibodies to the antigenic proteins MPB70 (where “MPB” is an acronym for protein from *M. bovis*^7^ and “70” refers to the protein’s relative electrophoretic mobility on a polyacrylamide gel^8^) and MPB83, which are typically produced in high amounts by *M. bovis*^7^.

The IDEXX ELISA has been tested on serum and milk samples from cattle that are not infected with *M. bovis*, and has been found to have a specificity of approximately 98%—that is, the test gives a positive result for only 2% of uninfected cattle^5^,^6^. The sensitivity of the ELISA has been evaluated using cattle that were verified to be infected with *M. bovis* using independent tests. Its sensitivity varied depending on the geographic origin of the infected cattle, ranging between 30% and 90%, and was approximately 62% overall^5^,^6^. The reasons for these marked geographic differences in test sensitivity are currently unknown.

In this study, we present additional data concerning the sensitivity of the IDEXX ELISA in different geographic regions, and show that the assay exhibits good sensitivity for serum samples from United Kingdom-derived cattle, moderate sensitivity for serum samples from United States-derived cattle, and low sensitivity for serum samples from Mexico-derived cattle. We then test the hypothesis that sequence variation in the *mpb70* and *mpb83* genes (including both their coding sequences and upstream and downstream regions), or in the genes whose corresponding proteins are involved in regulating their expression (*sigK* and *rskA*)^9^,^10^,^11^,^12^, is responsible for these geographic disparities. (See Supplementary Discussion S1 for further information on MPB70 and MPB83, the genes that encode them, and the proteins involved in regulating their expression). This hypothesis was investigated by obtaining whole genome shotgun (WGS) sequences from a large number of *M. bovis* strains from infected cattle in the United Kingdom, the United States, and Mexico and comparing their *mpb70*, *mpb83*, *sigK* and *rskA* sequences. Based on attributes of the sequences of these genes (see Supplementary Discussion S1), we searched for specific mutations that could explain false negatives by the IDEXX ELISA (for more information on these mutations, see Supplementary Discussion S2). If a sequence-based explanation exists for the geographic disparity in IDEXX ELISA sensitivity, then it would be expected that mutations like those described in Supplementary Discussion S2 would be found with the greatest frequency in *M. bovis* strains from Mexico, with a lower frequency in strains collected in the United States, and with the lowest frequency in strains from the United Kingdom.

## Methods

### Serum samples

A panel consisting of 400 bovine sera obtained from the United States Department of Agriculture (USDA) National Veterinary Services Laboratories (NVSL) Bovine Tuberculosis Serum Bank was tested with the IDEXX ELISA. These sera were from naturally infected cattle originating from the United States (*n* = 146), the United Kingdom (*n* = 126) or Mexico (*n* = 128). *M. bovis* infection was confirmed by the presence of visible lesions and bacterial culture.

### Enzyme-linked immunosorbent assays

A commercial ELISA, the *M. Bovis* Antibody Test Kit (IDEXX Laboratories, Westbrook, ME), was used to test all serum samples examined in this study. The kit contains all of the reagents required to perform the test, including: antigen-coated 96-well plates (16-well × 6 strips); positive and negative control sera; an anti-bovine IgG-horseradish peroxidase conjugate; 3,3′,5,5′-tetramethylbenzidine (TMB) substrate; a stop solution; a sample diluent, and a wash buffer. The assay and results interpretation were performed according to the manufacturer’s protocol. The antigen-coated plate and all reagents were warmed to room temperature (18–26 ^o^C) for at least two hours prior to use. The serum samples and control sera were each tested in duplicate adjacent wells (100 μL/well); all incubation steps were performed at room temperature and the plate was washed (300 μL/well) four times between reaction steps as indicated using a BioTek ELx405 plate washer (BioTek Instruments, Winooski, VT). The control and test sera were diluted 1:50 in the sample diluent, dispensed into the antigen-coated wells, and the plate was covered and incubated for 60 ± 5 min. Following a wash cycle, the conjugate was dispensed (100 μL/well) and the plate covered and incubated for 30 ± 2 min. Following a wash cycle, the TMB substrate was dispensed (100 μL/well) and the plate covered and incubated for 15 ± 1 min. The stop solution was then added (100 μL/well) and the absorbance measured using a Titertek Multiskan® MCC/340 MK II spectrophotometer (Flow Laboratories, Mississauga, ON) at a wavelength of 450 nm using an air blank.

The results for samples contained on a plate were considered valid only if the mean optical density (OD) values of the positive and negative controls on that plate were ≥0.300 and ≤0.200, respectively. The results are expressed as a Sample to Positive (S/P) ratio, which is calculated for each sample according to the following equation: S/P = (Mean Sample OD_450_ −Mean Negative Control OD_450_)/(Mean Positive Control OD_450_ −Mean Negative Control OD_450_).

A sample is scored positive if S/P ≥0.30.

### *M. bovis* DNA sequence data

**Figure 1:**
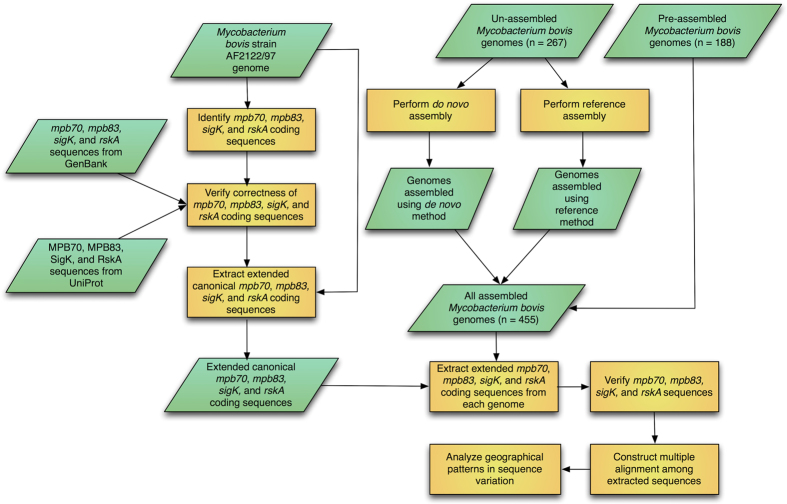
Summary of the methods used in this study. Green parallelograms represent inputs and outputs (data), while orange rectangles represent actions.

DNA sequence data for *M. bovis* strains isolated from infected cattle were primarily obtained from two sources: the Animal and Plant Health Agency (APHA) in the United Kingdom, and the Animal and Plant Health Inspection Service (APHIS) at the USDA. All strains sequenced by APHA were isolated from cattle that originated from, and were slaughtered in, the United Kingdom. Strains sequenced by APHIS were isolated from cattle that originated from either Mexico or the United States, with all cattle slaughtered in the United States. Cattle from Mexico were provided in association with the Secretariat of Agriculture, Livestock, Rural Development, Fisheries and Food (SAGARPA). Information about the strains sequenced by APHIS was provided as sample codes, most of which were of the form “StrainName_LocationOfOrigin_LocationOfSlaughter_HostType”. For example, the sample code “02–4414_SON_TX_Fed” indicates that *M. bovis* strain 02–4414 was isolated from an animal that originated from the Mexican state of Sonora and was fed and slaughtered in Texas. Although it is difficult to know where a particular animal became infected, for the purposes of this study the location of origin (Mexico in the above example) was used as the geographic location corresponding to a given strain. Some sample codes were of the form “StrainName_Location_HostType”; in these cases, the given location represented both the location of origin and the location of slaughter. Samples whose code contained “UNK” (unknown) as the location of origin were not used. One additional strain (*M. bovis* strain AF2122/97), which was sequenced by the Wellcome Trust Sanger Institute, was used; this strain was isolated from a cow in the United Kingdom and was the first *M. bovis* strain sequenced^13^. A graphical summary of all analyses performed for this study is shown in Fig. 1. A total of 12 sequenced *M. bovis* strains were isolated from cattle on which the IDEXX ELISA was performed, seven of which were ELISA-positive and five of which were ELISA-negative, allowing a direct relationship between genetic variation and ELISA result to be obtained for these strains.

### Assembly of *M. bovis* DNA sequence data

The genome of *M. bovis* strain AF2122/97 was downloaded fully assembled. Some of the genomes obtained from APHIS were already assembled by the Pathosystems Resource Integration Center (PATRIC)^14^,^15^,^16^. The remaining sequence data from APHIS, as well as all of the sequence data from APHA, required assembly, as they were in the form of raw sequence reads in FASTQ format (paired-end reads generated by the Illumina MiSeq platform). As a quality-control measure, two independent methods were used to assemble these reads. The first method (“*de novo* assembly”) involved using the ABySS program (version 1.5.2), which is designed to assemble short paired-end reads without the aid of a reference genome^17^. Due to the large number of genomes that had to be assembled, no manual finishing steps were performed after assembly by ABySS. In the second method (“reference assembly”), reads were mapped against a reference genome, and single nucleotide polymorphisms (SNPs) and insertions/deletions (indels) were detected from the resulting mapping. The genome of *M. bovis* strain AF2122/97 was used as the reference genome, as it is the most well-characterized *M. bovis* genome. Reference assembly was performed according to the best practices recommended by the authors of the Genome Analysis Toolkit (GATK)^18^. Specifically, the BWA program^19^ was used to map reads against the genome of *M. bovis* strain AF2122/97, resulting in a file of aligned reads in sequence alignment/map (SAM) format^20^. Utilities in the SAMtools package^20^ were used to convert the SAM file into the binary alignment/map (BAM) format, sort the BAM file, and index the BAM file for subsequent processing. Duplicate reads were detected and marked using the “MarkDuplicates” feature of Picard (http://broadinstitute.github.io/picard). The “RealignerTargetCreator” and “IndelRealigner” functions of GATK^21^,^22^ were used to improve the alignment of the reads. The “BaseRecalibrator” and “PrintReads” GATK functions were then used to calibrate the base-quality scores based on known high-quality SNPs. The GATK function “HaplotypeCaller” was used to generate a variant call format (VCF)^23^ file, which enumerates the SNPs and indels detected. Finally, the “FastaAlternateReferenceMaker” function was used to generate a FASTA file containing the same sequence as the reference genome, except with the variants specified in the VCF file.

### Identification of known *mpb70*, *mpb83*, *sigK*, and *rskA* coding sequences

To identify the coding sequence of *mpb70*, the list of genes in *M. bovis* strain AF2122/97 was searched for “mpb70”, and the corresponding coding sequence was extracted. To verify that this coding sequence was correctly annotated, GenBank^24^ was searched using the query “(mpb70) AND *mycobacterium bovis*[Organism]”, and the *mpb70* coding sequence was downloaded for four additional *M. bovis* strains. A multiple alignment of all five sequences was created using the program MUSCLE^25^,^26^ to confirm that all of the sequences represented the same gene. To further verify that the coding sequences were of *mpb70*, UniProt^27^ was searched using the query ‘mpb70 AND organism:“*Mycobacterium bovis* [1765]” (where 1765 is the taxonomic identifier of *M. bovis*), and the protein sequences of MPB70 (from the same *M. bovis* strains as for the coding sequences, where possible) were downloaded. The coding sequences identified earlier were translated into protein sequences using the European Molecular Biology Open Software Suite (EMBOSS)^28^ program transeq, and it was verified that the translation of the coding sequence for a given strain was identical to the protein sequence from the same strain downloaded from UniProt.

If the *mpb70* coding sequence from *M. bovis* strain AF2122/97 was found to be correct, then for the purposes of subsequent analyses, it was chosen to be the “canonical” *mpb70* coding sequence. As the regulatory elements surrounding the *mpb70* coding sequence were also of interest, a region consisting of the canonical *mpb70* coding sequence plus 500 base pairs (bp) upstream and 500 bp downstream was extracted from the genome of *M. bovis* strain AF2122/97. This will be referred to as the “extended canonical” *mpb70* coding sequence. This sequence was then used to identify the *mpb70* coding sequence and surrounding bases in each of the *M. bovis* genomes (see the following section for details).

The same procedure as described above was used to verify the correctness of the *mpb83*, *sigK*, and *rskA* coding sequences in *M. bovis* strain AF2122/97 and to extract the extended canonical coding sequence for each gene.

### Identification of *mpb70*, *mpb83*, *sigK*, and *rskA* extended coding sequences in the *M. bovis* genomes

The extended canonical *mpb70* coding sequence, which was identified as described above, was used to extract the extended *mpb70* coding sequence from the genome of each of the *M. bovis* strains examined. The simplest way to do this would be to use the EMBOSS program needle^29^ to perform a global alignment between the extended canonical coding sequence and each of the contigs that comprise a given genome. However, because the contigs may be very large, needle may either take a very long time to run or exceed the computer’s memory capacity. To solve this problem, the following procedure was used. For a given strain, a Basic Local Alignment Search Tool (BLAST)^30^,^31^ search was performed using the extended canonical *mpb70* coding sequence as the query and the assembled genome of that strain as the database. From the local alignment reported by BLAST, the identity of the contig containing *mpb70* was determined, as well as the approximate region of that contig that contained the extended *mpb70* coding sequence. The region surrounding the local alignment (specifically, 2000 bp upstream of the beginning of the alignment to 2000 bp downstream of the end of the alignment) was extracted using the EMBOSS program seqret. A global alignment was then performed between the extended canonical *mpb70* coding sequence and the region extracted using seqret. The portion of the extracted region that aligned with the extended canonical *mpb70* coding sequence was determined, and after removing any gap characters, the corresponding sequence was written to a FASTA file. The same procedure was used to extract the extended coding sequences for *mpb83*, *sigK*, and *rskA* from each genome.

### Verification of *mpb70*, *mpb83*, *sigK*, and *rskA* extended coding sequences

After extracting the *mpb70*, *mpb83*, *sigK*, and *rskA* extended coding sequences from each of the *M. bovis* genomes, their correctness was evaluated. This was done using three different methods. The first method was applied only to genomes that were assembled by the authors of this study, while the other two methods were applied to all of the genomes.

- The extended coding sequences of *mpb70*, *mpb83*, *sigK*, and *rskA* were extracted from both the *de novo* assembly and the reference assembly of a given genome, and the sequence of a given gene derived from the *de novo* assembly was compared with the sequence of the same gene derived from the reference assembly. The two sequences were then checked to ensure that they were identical. If the sequences were found to be different, then the source of the discrepancy was ascertained by examining an alignment between the two sequences, and by manually examining reads corresponding to the discrepant portion of the gene. Using this information, the assembly method that appeared most likely to be correct was identified.
- The length of each extended coding sequence was compared to that of the corresponding extended canonical coding sequence. If the lengths differed, then a pairwise global alignment between the two sequences was examined to determine whether the difference in lengths appeared to be due to true insertions or deletions, or due to sequencing or assembly errors.
- Each extended coding sequence was searched for the presence of ambiguous nucleotides (represented as the letter “N”). If a sequence contained one or more ambiguous nucleotides, then it was aligned with the corresponding extended canonical coding sequence to determine whether the presence of ambiguous nucleotides coincided with further divergence from the extended canonical coding sequence. If so, then it is likely that those differences represent sequencing errors rather than true sequence variations.

### Comparison of *mpb70*, *mpb83*, *sigK*, and *rskA* extended coding sequences from the United Kingdom, the United States, and Mexico

For each gene (*mpb70*, *mpb83*, *sigK*, and *rskA*), the program MUSCLE^25^,^26^ was used to construct a multiple alignment of the extended coding sequences corresponding to that gene. The alignments were visualized using Jalview^32^,^33^, and variable positions in the alignment were identified and summarized. Any geographic patterns in the detected variation were then identified. For the 12 *M. bovis* strains for which both a sequenced genome and an IDEXX ELISA result were available, we investigated whether there was any relationship between the result of the IDEXX test and the sequences of *mpb70*, *mpb83*, *sigK*, and *rskA* in a given strain’s genome.

### Whole-genome phylogenetic analysis

A whole-genome phylogenetic analysis was performed on the set of 12 *M. bovis* genomes that were isolated from cattle on which the IDEXX ELISA was performed. Raw FASTQ files were analyzed using the NVSL in-house pipeline (see https://github.com/USDA-VS). Briefly, reads were aligned to the genome of *M. bovis* strain AF2122/97 using BWA^19^, SAMtools^20^, and Picard (http://broadinstitute.github.io/picard). BAM files were processed using the GATK “best practices” workflow. SNPs were called using GATK’s “UnifiedGenotyper” function, outputting SNPs to VCF files^18^,^21^,^22^. Results were filtered using a minimum QUAL score of 150 and AC = 2. From the VCF files, SNPs gathered were outputted in three formats: an aligned FASTA file, a tab-delimited file with the position location and SNPs grouped and sorted, and a phylogenetic tree created with RAxML^34^. SNPs were visually validated using Integrative Genomics Viewer^35^.

## Results

### Enzyme-linked immunosorbent assays

**Figure 2:**
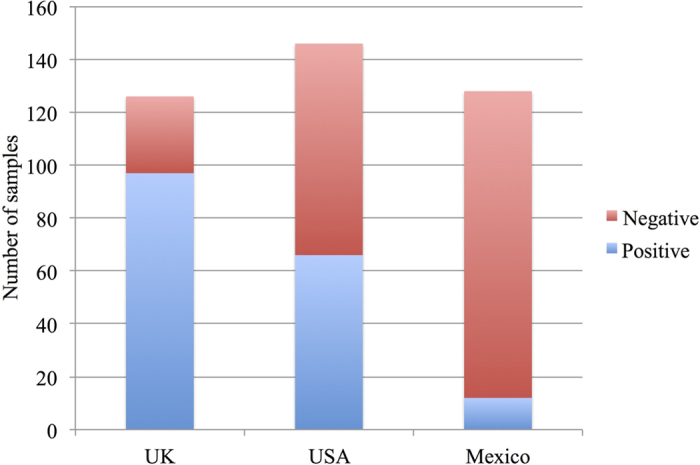
Serum samples. Number of serum samples from *M. bovis*-infected cattle that tested positive or negative according to the IDEXX ELISA in the United Kingdom, the United States, and Mexico.

The IDEXX ELISA was performed on 126 bovine serum samples from the United Kingdom, 146 samples from the United States, and 128 samples from Mexico, all of which were derived from cattle with a confirmed *M. bovis* infection, associated with grossly visible lesions and a positive bacterial culture. Despite a confirmed *M. bovis* infection in all cattle sampled, the sensitivity of the ELISA varied greatly depending on the geographic location of the infected animal, with sensitivities of 77%, 45%, and 9% in the United Kingdom, the United States, and Mexico, respectively (Fig. 2). A manuscript describing the results of these tests in further detail is currently in preparation.

### *M. bovis* DNA sequence data

DNA sequence data for different *M. bovis* strains were obtained primarily from APHIS and APHA, with one strain having been sequenced by the Wellcome Trust Sanger Institute. These strains were isolated from cattle that originated from the United Kingdom, the United States, or Mexico. The 34 WGS sequences from the United Kingdom were chosen to represent the total diversity found in a collection of over 700 isolates from the United Kingdom for which WGS data are available and represent genotypes that are geographically localized to the United Kingdom. Table 1 summarizes the number of genomes analyzed from each country of origin and each sequencing center. Supplementary Table S1 contains a complete list of the *M. bovis* strains used in this study.

### Assembly of *M. bovis* DNA sequence data

Of the 455 *M. bovis* genomes analyzed, 188 were obtained in an already-assembled form. Of these, 187 were assembled using a pipeline developed by PATRIC^14^,^15^,^16^, while the other was assembled by the Wellcome Trust Sanger Institute using the Phrap program^13^,^36^. The remaining 267 genomes were obtained as unassembled reads. These genomes were assembled by the authors of this study using two separate methods: a “*de novo* assembly” using the ABySS assembler^17^, and a “reference assembly”, which was performed by mapping reads against a reference genome (*M. bovis* strain AF2122/97), detecting SNPs and indels, and then inferring the new genome sequence based on the detected changes to the sequence of the reference genome.

The soundness of each assembly was evaluated based on the number of contigs generated and the aggregate number of base pairs in those contigs. The genome of *M. bovis* strain AF2122/97 contains approximately 4.3 Megabase pairs (Mbp); as extensive finishing was performed on this genome sequence, it contained just a single contig (representing *M. bovis*’s sole chromosome). The number of contigs in the genomes assembled using PATRIC’s pipeline ranged from 83 to 2,504, and the lengths of most of them (173 out of 187) were between 4 and 5 Mbp. The remaining genomes had greater lengths, ranging from 5 Mbp to 13 Mbp, likely due to errors in the assembly process. The sizes of the *de novo* assemblies generated by the authors of this study were similar to those of the genomes assembled by PATRIC. Specifically, 252 out of 267 genomes were between 4 and 5 Mbp in length, while the remaining 15 ranged between 5 and 17 Mbp. The number of contigs ranged from 221 to 14,410, with most genomes being comprised of fewer than 1,000 contigs. Due to the way they were constructed, all of the reference assemblies consisted of a single contig, and their lengths were all very similar to that of *M. bovis* strain AF2122/97. The shortest reference assembly and the longest reference assembly differed in length by fewer than 6,000 bp.

It should be noted that the genomes of the different *M. bovis* strains almost certainly do not vary to the extent suggested above (e.g., varying in length between 4 and 5 Mbp); the level of variation observed is likely a consequence of an imperfect assembly process wherein no manual joining of contigs or other finishing steps were performed. Lack of finishing did not represent a problem in this study, as the four genes of interest were successfully extracted from over 96% of the assembled genomes (see subsequent sections).

### Identification of known *mpb70*, *mpb83*, *sigK*, and *rskA* coding sequences

The procedure described in Methods was used to determine whether the sequence annotated as *mpb70* in the genome of *M. bovis* strain AF2122/97 actually represented *mpb70*. It was determined that this sequence was correct, as it was consistent with the sequences annotated as *mpb70* from other *M. bovis* strains downloaded from GenBank, and its translation was consistent with several MPB70 protein sequences retrieved from UniProt. Thus, the *mpb70* coding sequence from *M. bovis* strain AF2122/97 was chosen to be the canonical *mpb70* coding sequence. The extended canonical *mpb70* coding sequence was obtained by extracting the sequence of *mpb70* from the genome of *M. bovis* strain AF2122/97 plus 500 bp upstream and 500 bp downstream. This sequence is shown in Supplementary Fig. S1.

The sequences annotated as *mpb83*, *sigK*, and *rskA* in the genome of *M. bovis* strain AF2122/97 were also found to be correct, and were thus chosen as the canonical coding sequences for these genes. As with *mpb70*, the extended canonical coding sequence, which consisted of the canonical coding sequence plus 500 bp upstream and downstream, was extracted for each of these genes.

### Identification of *mpb70*, *mpb83*, *sigK*, and *rskA* extended coding sequences in the *M. bovis* genomes

The extended coding sequences of *mpb70*, *mpb83*, *sigK*, and *rskA* were each found in all of assembled *M. bovis* genomes except the *de novo* assembly of *M. bovis* strain 00–5477, in which none of the four genes were found. This is likely because the *de novo* assembly of this strain was poor. The sequences for this strain were thus extracted from the reference assembly. A small number of extended coding sequences were found in an incomplete form; more details are given in the following section.

### Verification of *mpb70*, *mpb83*, *sigK*, and *rskA* extended coding sequences

As described in Methods, three different methods were used to evaluate the validity of the extended *mpb70*, *mpb83*, *sigK*, and *rskA* coding sequences that were extracted from the genomes of the different *M. bovis* strains. The first method applied only to the strains whose genomes were assembled by the authors of this study, and involved comparing the extended coding sequence of a given gene extracted from the *de novo* assembly of a given strain’s genome with the extended coding sequence of the same gene extracted from the corresponding reference assembly. Of the 267 strains whose genomes were assembled by the authors, the extended *mpb70* coding sequence extracted from the *de novo* assembly was identical to that extracted from the reference assembly for 253 of them. The concordance of these sequences was a strong indicator that they were correct and error-free. In the other 14 strains, the sequence of the extended *mpb70* coding sequence derived from the *de novo* assembly was found to differ by at least one mismatch or indel relative to the sequence derived from the reference assembly. To examine the reasons for these discrepancies, the two sequences for each strain were globally aligned with one another using needle, and the alignments were manually examined to determine which assembly process (*de novo* or reference) produced the correct sequence. This determination was sometimes aided by examining the original sequence reads. In most cases, the discrepancy was due to either incorrectly-mapped reads in the reference assembly (in which case the *de novo* assembly was deemed to be correct) or to the absence of a contig in the *de novo* assembly that contained the entirety of the extended *mpb70* coding sequence (in which case the reference assembly was deemed to be correct). The number of strains for which the extended coding sequence of a given gene was not identical in the *de novo* and reference assemblies was 21, 9, and 10 for *mpb83*, *sigK*, and *rskA*, respectively. The reasons for the observed discrepancies were similar to those for *mpb70*. Over all 54 discrepancies, the reference assembly was found to contain the correct sequence for 29 of the cases versus 25 for the *de novo* assembly. A list of strains for which discrepancies were observed for each gene, as well as descriptions of the alignment and the cause of the discrepancy, is given in Supplementary Table S2.

After evaluating the consistency of the extended coding sequences derived from the *de novo* and reference assemblies, the correctness of the sequences for all 455 strains was evaluated by identifying those that were either of anomalous length (that is, the length of a given extended coding sequence differed from the corresponding sequence in *M. bovis* strain AF2122/97) or contained ambiguous nucleotides. Any extended coding sequence that fell into at least one of these two categories was aligned to the corresponding extended canonical coding sequence from *M. bovis* strain AF2122/97. Interestingly, the set of sequences containing ambiguous nucleotides was almost identical to the set of sequences having lengths different than the corresponding sequence in *M. bovis* strain AF2122/97. This was because ambiguous nucleotides almost always coincided with multiple insertions or deletions nearby in the alignment. These indels almost certainly represent sequencing errors, rather than real biological variation. Thus, such sequences were not included in subsequent analyses. Table 2 contains the number of genomes from each geographic region from which the extended coding sequence of a given gene was retained. Supplementary Table S3 contains detailed information about each extended coding sequence that contained indels (relative to the corresponding extended canonical coding sequence) or ambiguous nucleotides.

### Comparison of *mpb70*, *mpb83*, *sigK*, and *rskA* extended coding sequences from the United Kingdom, the United States, and Mexico

To determine whether sequence variation in one or more of the genes analyzed in this study may explain geographic differences in IDEXX ELISA sensitivity, a multiple alignment was created for each gene consisting of all the corresponding extended coding sequences from the *M. bovis* genomes (except those that were discarded, as described above). From each multiple alignment, the variation in the extended coding sequences was analyzed, and the existence of any geographic patterns was ascertained. For the 12 *M. bovis* strains that were isolated from cattle on which the IDEXX ELISA was performed, we directly correlated sequence variation with the success or failure of the IDEXX ELISA.

Variation in *mpb70*—A complete description of the genetic variation in the extended *mpb70* coding sequences is given in Supplementary Table S4. The entire alignment contained 1,582 positions, 22 of which were variable. Six of the variable positions were upstream of the coding sequence, six were in the coding sequence itself, and 10 were downstream.

With respect to the mutations outlined in Supplementary Discussion S2 that would be of particular interest, none of the variable positions that were upstream of the coding sequence were found within the −10 or −35 promoter regions. (In the subsequent discussion, numbers prefixed with a minus sign or a plus sign indicate positions upstream or downstream of the coding sequence, respectively, while numbers with no prefix indicate positions within the coding sequence. For example “−1” means the base immediately preceding the start of the coding sequence, while “1” means the first base of the coding sequence and “+1” means the base immediately after the stop codon). Four of the six variable positions in the coding sequence were in the portion corresponding to the signal peptide, although the significance of this with respect to the antigenic properties of MPB70 is unclear.

Of the 451 *M. bovis* genomes from which an extended *mpb70* coding sequence was extracted, 375 genomes (83.1%) contained exactly the same sequence, including that of *M. bovis* strain AF2122/97. The percentage of *M. bovis* genomes that contained this common sequence was relatively constant among the geographic regions (Table 3). Of the seven sequenced *M. bovis* strains for which the IDEXX ELISA was able to successfully detect antibodies, the genomes of five contained the same *mpb70* sequence as that of strain AF2122/97 (Table 4). The genomes of three out of five IDEXX-negative strains contained the same *mpb70* sequence as that of strain AF2122/97.

Most of the extended *mpb70* coding sequences that were not identical to the common sequence differed by only a single nucleotide; three differed by two nucleotides and one differed by three. One sequence contained a deletion relative to the common sequence. One variant (G → A in position −54) was by far the most common, with 55 sequences containing only this mutation and two additional sequences containing this mutation plus others. Supplementary Fig. S2 contains the complete multiple alignment among all 451 extended *mpb70* coding sequences.

Variation in *mpb83*—Supplementary Table S5 describes the genetic variation in the extended *mpb83* coding sequences. Although the alignment length (1,663 positions) was similar to the alignment for *mpb70* (1,582 positions), *mpb83* contained fewer than half the number of variable positions (22 versus 10, respectively). Four of the 10 positions were upstream of the coding sequence, while three were within the coding sequence and three were downstream.

As with *mpb70*, none of the variable positions that were found in the upstream region occurred near the promoter. One of the variable positions in the coding sequence was within the portion corresponding to the signal peptide, while the other two were in the region corresponding to the mature protein.

Of the 438 extended *mpb83* coding sequences analyzed, 372 of the sequences were identical to one another. The sequence of *M. bovis* strain AF2122/97 was among these. Of the seven IDEXX ELISA-positive strains, the genomes of six contained the same *mpb83* sequence as that of strain AF2122/97, while the same was true for three of the five IDEXX-negative strains (Table 4).

Table 3 shows that the proportion of *M. bovis* genomes that contained the common sequence was greater than 80% in all three countries. Nearly all of the sequences that differed from this common sequence did so by a single mutation. None of the sequences contained an insertion or deletion relative to that of *M. bovis* strain AF2122/97. The most common variant was T → G in position +193. The complete multiple alignment of the extended *mpb83* coding sequences is shown in Supplementary Fig. S3.

Variation in *sigK*—The genetic variation in the extended *sigK* coding sequences is shown in Supplementary Table S6. There were 11 variable positions in the 1,564-position alignment. Nine of these positions were in the downstream portion of the sequence, while two were in the coding sequence and none were in the upstream region.

As described in Supplementary Discussion S1, it has been found that certain strains of *M. bovis* express *mpb70* and *mpb83* in very low amounts due to a mutation in the start codon of *sigK*. However, none of the genomes analyzed in this study contained such a mutation.

The extended *sigK* coding sequences were even more homogeneous than those of *mpb70* and *mpb83*. Specifically, 419 of the 440 *sigK* sequences examined (95.2%) were identical to one another. This common sequence was also found in *M. bovis* strain AF2122/97. The percentage of *M. bovis* genomes that contained the common sequence was very high (over 93%) in all three countries (Table 3). All of the IDEXX ELISA-positive strains contained the same *sigK* sequence as that of strain AF2122/97, while the same was true for four of the five negative strains (Table 4).

Two variants (T → G in position 474 and T → C in position +49) were present in several genomes. The T → G mutation in position 474 is a synonymous substitution (this mutation is in the third position of the codon GT*, and both GTT and GTG code for valine). Supplementary Fig. S4 contains the complete multiple alignment of the extended *sigK* coding sequences.

Variation in *rskA*—Supplementary Table S7 shows the variation found in the extended *rskA* coding sequences. Fourteen of the 1,699 alignment positions were variable. The number of variable positions that were upstream of the coding sequence, within the coding sequence, or downstream of the coding sequence was four, eight, and two, respectively. Note that because of the close proximity of *sigK* and *rskA* in the *M. bovis* genome, there is some overlap between the positions in Supplementary Table S6 and those in Supplementary Table S7. For example, position −134 in the extended *rskA* coding sequence is the same as position 474 in the extended *sigK* coding sequence.

In Supplementary Discussion S2, it is suggested that the mutations D107G and E184G in RskA could render it functional, promoting the negative regulation of *mpb70* and *mpb83* expression. However, none of the genomes contained either mutation.

The extended *rskA* coding sequences were only slightly less homogeneous than those of *sigK*, with 416 of 440 sequences (94.5%) being identical to one another (and to that of *M. bovis* strain AF2122/97). The percentages of *M. bovis* genomes that contained the common *rskA* sequence in a given country were also similar to *sigK* (Table 3). Each of the seven IDEXX ELISA-positive strains and four of the five negative strains contained the same *rskA* sequence as *M. bovis* strain AF2122/97 (Table 4).

Common variants were T → G in position −134 and T → C in position 6. (These are the same variants as shown in Supplementary Table S6 for *sigK*, except the positions are relative to the beginning of the *rskA* coding sequence). The T → C mutation in position 6 is a synonymous substitution (this mutation is in the third position of the codon AC*, and both ACT and ACC code for threonine). The complete multiple alignment of the extended *rskA* coding sequences is shown in Supplementary Fig. S5.

### Whole-genome phylogenetic analysis

**Figure 3:**
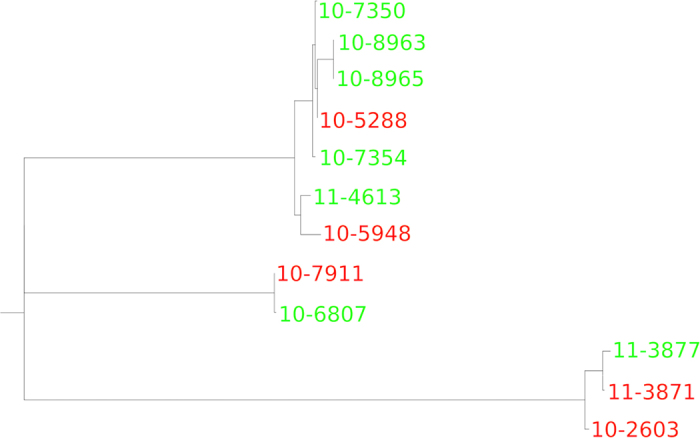
Whole-genome phylogenetic analysis of 12 *M. bovis* strains isolated from cattle on which the IDEXX ELISA was performed. Green strains are those that tested positive, while red strains tested negative.

In addition to examining sequence variation in specific genes (*mpb70*, *mpb83*, *sigK*, and *rskA)*, we also performed a whole-genome phylogenetic analysis on the set of 12 sequenced *M. bovis* strains that were isolated from cattle on which the IDEXX ELISA was performed. A maximum likelihood phylogenetic tree representing the overall genomic relatedness of these strains is shown in Fig. 3. The tree appears to exhibit no phylogenetic clustering among strains with the same IDEXX ELISA outcome, with several branches containing some strains that tested positive and some that tested negative.

## Discussion

As described earlier, the goal of this study was to test the hypothesis that geographic differences in the sensitivity of the IDEXX ELISA in the United Kingdom, the United States, and Mexico could be explained by sequence variation in *mpb70* and *mpb83*, which encode the two proteins used in the assay, or in *sigK* and *rskA*, which encode proteins regulating the expression of *mpb70* and *mpb83*. To this end, the extended coding sequence of each gene was extracted from the genome sequences of 455 *M. bovis* strains, 34 of which were isolated from infected cattle present in the United Kingdom, 233 of which were isolated from infected cattle originating from the United States, and 188 of which were isolated from infected cattle originating from Mexico (Table 1). Due to errors in sequence assembly or the original sequence data, a correct and complete sequence for each gene could not be obtained from some genome sequences. However, sequences were obtained from at least 96% of the genomes for all four genes (Table 2), providing sufficient data to analyze sequence variation in each gene and determine if there were geographic patterns in that variation.

The data presented suggest that sequence variation in the *mpb70*, *mpb83*, *sigK*, and *rskA* genes does not explain geographic differences in IDEXX ELISA sensitivity. Specifically, the observations that support this conclusion are as follows.

- Observation #1: For each gene, a single conserved sequence was found in a large proportion (at least 83%) of the *M. bovis* genomes (Table 3).
- Observation #2: The common sequence of all four genes was also present in the genomes of the majority of both the 7 IDEXX ELISA-positive strains and the 5 ELISA-negative strains (Table 4).
- Observation #3: The percentage of *M. bovis* genomes where the sequence of a given gene differed from the common sequence (and thus potentially might not be detected by the IDEXX ELISA) in a given country was inconsistent with the IDEXX ELISA sensitivity in that country (Fig. 2 and Table 3). For instance, the genomes of 89.9% of the *M. bovis* strains from Mexico contained the most common extended *mpb70* coding sequence, which was detectable by the IDEXX ELISA (observation #2). However, the sensitivity of the IDEXX ELISA was reported to be less than 10% in Mexico. Thus, it appears that sequence variation in the *mpb70* gene cannot explain the low sensitivity of the IDEXX ELISA in Mexico. A similar argument applies to the other three genes.
- Observation #4: In the sequences that differed from the common sequence of each gene, the positions of the variations did not correspond to regions that would be expected to affect the sensitivity of the IDEXX ELISA. In Supplementary Discussion S2, a number of potential mutations are described that could potentially prevent detection of *M. bovis* infection on the basis of antibody responses to the MPB70 and MPB83 proteins. However, none of these mutations were detected in the sequences examined.

In addition, the results of the whole-genome phylogenetic analysis suggest that, beyond *mpb70*, *mpb83*, *sigK*, and *rskA*, sequence variation in other regions of the genome do not appear to play a role in influencing the sensitivity of the IDEXX ELISA, as strains that were positive according to this assay shared branches of the phylogenetic tree with strains that tested negative (Fig. 3).

For the IDEXX ELISA to successfully identify an *M. bovis* infection, the antibodies from the serum sample must interact with the versions of MPB70 and MPB83 used in the assay. Thus, the overall accuracy of the test relies heavily on the level of sequence variation in *mpb70* and *mpb83*. Specifically, if the degree of sequence variation in *mpb70* and *mpb83* was too great, then the antibodies produced by some infected cows may not interact with the versions of MPB70 and MPB83 used in the IDEXX ELISA. The accuracy of the test also relies on MPB70 and MPB83 being produced in sufficient quantity throughout infection to induce and maintain a detectable level of specific antibodies circulating in blood. Therefore, a high level of sequence variation in the regulators of *mpb70* and *mpb83* expression (*sigK* and *rskA)* could also reduce the sensitivity of the test, as some *M. bovis* genomes could contain versions of these genes that prevent MPB70 and MPB83 from being produced, and thus no antibody response would be generated. As described above, however, the level of sequence variation in *mpb70*, *mpb83*, *sigK*, and *rskA* is very low. This suggests that MPB70 and MPB83 are appropriate choices as target antigens for use in an ELISA.

Given that sequence variation in the *mpb70, mpb83, sigK,* and *rskA* genes does not appear to explain the geographic variations in IDEXX ELISA sensitivity, other possible explanations need to be explored. In the original paper describing testing of the IDEXX ELISA, two major factors were mentioned that appeared to impact assay sensitivity^5^. First, the performance of a skin test (the injection of *M. bovis* purified protein derivative [PPD] to measure delayed-type hypersensitivity reactions) greatly increased IDEXX ELISA sensitivity several days after PPD was injected into infected cattle. Second, the stage of disease appeared to play a role, with greater duration of infection also associated with higher test sensitivity. Thus, either of these factors may have contributed to the differences in IDEXX ELISA sensitivity observed in this study. All serum samples were, however, collected from animals with visible lesions from which *M. bovis* was cultured. Figure 3 shows that animals infected with *M. bovis* strains that are similar at the whole-genome level can test either positive or negative with the IDEXX ELISA. We cannot, however, exclude the possibility that genetic variation in portions of the *M. bovis* genome not examined in the present study might play a role in regulating MPB70 and MPB83 protein expression. Regulatory mechanisms that alter the abundance, stability, or translation of *mpb70* and *mpb83* transcripts, such as siRNAs or miRNAs, could also play a role. As future work, RNA-seq or qRT-PCR analysis could be used to determine whether differences in *mpb70* and *mpb83* transcript abundance are observed in *M. bovis* strains isolated from IDEXX ELISA-positive versus -negative animals. This analysis would determine whether further investigations are warranted to determine if genetically encoded differences in non-coding RNAs regulate *mpb70* and *mpb83* transcription or translation.

## Additional Information

**How to cite this article**: Trost, B. *et al.* Investigation of the cause of geographic disparities in IDEXX ELISA sensitivity in serum samples from *Mycobacterium bovis*-infected cattle. *Sci. Rep.* **6**, 22763; doi: 10.1038/srep22763 (2016).

## Supplementary Material

Supplementary Information

Supplementary Table S1

Supplementary Table S2

Supplementary Table S3

## Figures and Tables

**Table 1:** Summary of *M. bovis* strains examined.

	APHIS	APHA	WTSI	Total
United Kingdom	0	33	1	34
United States	233	0	0	233
Mexico	188	0	0	188
Total	421	33	1	455

The number of strains from each country of origin (United Kingdom, United States, and Mexico) and sequencing group (Animal and Plant Health Inspection Service (APHIS), Animal and Plant Health Agency (APHA), and Wellcome Trust Sanger Institute (WTSI)) are indicated.

**Table 2:** Number of *M. bovis* genomes from each region from which a complete and error-free extended coding sequence of a given gene could be extracted.

	*mpb70*	*mpb83*	*sigK*	*rskA*
United Kingdom	34	34	34	34
United States	229	216	222	222
Mexico	188	188	184	184
Total	451	438	440	440

The total number of genomes from the United Kingdom, the United States, and Mexico was 34, 233, and 188, respectively (see Table 1).

**Table 3:** Percentage of genomes from each country for which the extended coding sequence of a given gene was 100% identical to the most common extended coding sequence.

	*mpb70*	*mpb83*	*sigK*	*rskA*
United Kingdom	94.1%	88.2%	94.1%	94.1%
United States	76.0%	80.1%	93.7%	93.2%
Mexico	89.9%	89.9%	97.3%	96.2%
Overall	83.1%	84.9%	95.2%	94.5%

The overall percentages are not simply the average of the other three percentages, but rather are weighted averages that take into account the number of sequences from each region.

**Table 4:** IDEXX ELISA status and sequence type of 12 *M. bovis* strains isolated from cattle.

Strain	IDEXX ELISA result	Same sequence as that of *M. bovis* strain AF2122/97?			
		*mpb70*	*mpb83*	*sigK*	*rskA*
10–6807	+	Yes	Yes	Yes	Yes
10–7350	+	Yes	Yes	Yes	Yes
10–7354	+	Yes	Yes	Yes	Yes
10–8963	+	Yes	Yes	Yes	Yes
10–8965	+	Yes	Yes	Yes	Yes
11–3877	+	No	No	Yes	Yes
11–4613	+	No	Yes	Yes	Yes
10–2603	−	No	No	No	No
10–5288	−	Yes	Yes	Yes	Yes
10–5948	−	Yes	Yes	Yes	Yes
10–7911	−	Yes	Yes	Yes	Yes
11–3871	−	No	No	Yes	Yes
