# Supplementary material for: Investigation of the cause of geographic disparities in IDEXX ELISA sensitivity in serum samples from Mycobacterium bovis-infected cattle
Source: Sci Rep. 2016 Mar 7;6:22763. doi: 10.1038/srep22763 (PMC4780098; doi:10.1038/srep22763)

Investigation of the cause of geographic disparities in IDEXX ELISA sensitivity in serum samples from Mycobacterium bovis-infected cattle

Brett Trost, Tod Stuber, Om Surujballi, Jeffrey Nelson, Suelee Robbe-Austerman,Noel H. Smith, Louis Desautels, Suresh K. Tikoo, and Philip Griebel

Supplementary Information

The following list describes each piece of supplementary information associated with this paper. All of the information is contained in this document except for Supplementary Tables 1, 2, and 3, which are provided separately in Microsoft Excel format. For Supplementary Figures S2-S5, you will have to zoom in significantly in your PDF reader in order for the detail in the figure to be legible. For the information contained in this document, the page number at which the information begins is given in parentheses; otherwise, the associated filename is given.

- Supplementary Discussion S1 (page 4): Detailed background information on *mpb70*, *mpb83*, *sigK*, and *rskA*.
- Supplementary Discussion S2 (page 13): Mutations in *mpb70*, *mpb83*, *sigK*, and *rskA* that could cause false negatives by the IDEXX ELISA.
- Supplementary Table S1 (supplementary_table_S1.xlsx): Complete list of *M. bovis* strains examined. Each row contains information for a single *M. bovis* strain. The first column contains the name of the strain, while the second column contains its sample code, if applicable. The third column contains the country of origin of the bovine from which the strain was isolated, while the fourth column indicates the organization that sequenced the genome of the strain.
- Supplementary Table S2 (supplementary_table_S2.xlsx): List of cases where the extended coding sequence of a given gene from the *de novo* assembly of a given *M. bovis* strain differed from that of the reference assembly for the same strain. The first column contains the strain for which the discrepancy occurred. The second column contains a description of the global alignment between the two sequences, while the third column describes the problem that appeared to cause the sequences to differ. The fourth

column indicates which assembly process (*de novo* or reference) appeared to contain the correct sequence.

- Supplementary Table S3 (supplementary_table_S3.xlsx): List of anomalous extended coding sequences. The first column contains the strain from which the extended coding sequence was extracted. The second column indicates whether or not the extended coding sequence contained ambiguous nucleotides. The third column contains a description of the global alignment between the extended coding sequence from that strain and the corresponding extended coding sequence from *M. bovis* strain AF2122/97. The fourth column indicates whether the sequence was used for subsequent analyses (retained) or discarded.

- Supplementary Table S4 (page 15): Sequence variation in the extended *mpb70* coding sequences. A multiple sequence alignment was constructed among the extended *mpb70* coding sequences from 451 *M. bovis* strains, and alignment positions that did not contain the same nucleotide in all strains were identified. The header row of the table contains the alignment positions that were variable. Numbers prefixed with a minus sign or a plus sign indicate positions upstream or downstream of the coding sequence, respectively, while numbers with no prefix indicate positions within the coding sequence. For example ?1 means the base immediately preceding the start of the coding sequence, while 1 means the first base of the coding sequence and +1 means the base immediately after the stop codon. The extended canonical *mpb70* coding sequence (i.e., the *mpb70* sequence from *M. bovis* strain AF2122/97) was used as a reference. Specifically, the first main row of the table shows the bases that occur in each variable position in the reference sequence. The remaining rows show mutations in a given strain relative to the reference strain. If the nucleotide in a given position was the same as in the reference strain, then a period is shown; otherwise, the nucleotide (or gap) at that position is indicated. Only strains whose extended *mpb70* coding sequence differed from that of *M. bovis* strain AF2122/97 are shown; the extended *mpb70* coding sequence for all strains not shown were 100% identical to that of *M. bovis* strain AF2122/97.

- Supplementary Table S5 (page 18): Sequence variation in the extended *mpb83* coding sequences. A multiple sequence alignment was constructed among the extended *mpb83* coding sequences from 438 *M. bovis* strains, and alignment positions that did not contain the same nucleotide in all strains were identified. For further details, see the caption describing Supplementary Table S4.

- Supplementary Table S6 (page 20): Sequence variation in the extended *sigK* coding sequences. A multiple sequence alignment was constructed among the extended *sigK* coding sequences from 440 *M. bovis* strains, and alignment positions that did not contain the same nucleotide in all strains were identified. For further details, see the caption describing Supplementary Table S4.

- Supplementary Table S7 (page 21): Sequence variation in the extended *rskA* coding sequences. A multiple sequence alignment was constructed among the extended *rskA* coding sequences from 440 *M. bovis* strains, and alignment positions that did not contain the same nucleotide in all strains were identified. For further details, see the caption describing Supplementary Table S4.

- Supplementary Figure S1 (page 23): The extended canonical *mpb70* coding sequence. The coding sequence is shown in blue, while the upstream and downstream regions (500 bp each) are shown in black.

- Supplementary Figure S2 (page 24): Multiple alignment of the extended *mpb70* coding sequences from 451 *M. bovis* genomes. The consensus sequence is shown at the bottom of the alignment. Nucleotides in the individual sequences that match the consensus sequence are shown in blue, while those that differ from the consensus sequence are shown in white. The coding sequence is indicated by a red box.

- Supplementary Figure S3 (page 25): Multiple alignment of the extended *mpb83* coding sequences from 438 *M. bovis* genomes. For further details, see the description of Supplementary Figure S2.

- Supplementary Figure S4 (page 26): Multiple alignment of the extended *sigK* coding sequences from 440 *M. bovis* genomes. For further details, see the description of Supplementary Figure S2.

- Supplementary Figure S5 (page 27): Multiple alignment of the extended *rskA* coding sequences from 440 *M. bovis* genomes. For further details, see the description of Supplementary Figure S2.

Supplementary Discussion S1: Detailed background information on *mpb70*, *mpb83*, *sigK*, and *rskA*

This document provides background information on the antigens used by the IDEXX ELISA—the proteins MPB70 and MPB83—as well as their corresponding genes (*mpb70* and *mpb83*). Specifically, their sequence characteristics are summarized in Section 1, while the three-dimensional structures of MPB70 and MPB83 are described in Section 2. Current knowledge concerning the functions of MPB70 and MPB83 is covered in Section 3, and Section 4 describes the existence of homologues of MPB70 and MPB83 in other bacteria. Background data are also given on two proteins and their corresponding genes that are known to regulate the expression of *mpb70* and *mpb83* (Section 5).

The information presented in this section comprises a mixture of results previously reported in the literature and simple *in silico* analyses performed by the authors of this study. Unless otherwise specified, the information is specific to *M. bovis* strain AF2122/97, which was the first *M. bovis* strain to have its genome sequenced.

1 Sequences of *mpb70*, *mpb83*, and their protein products

Including the stop codon, the coding sequence of *mpb70* is 582 bp in length, and the MPB70 protein thus contains 193 amino acid residues. The first 30 residues of MPB70 constitute a signal peptide that is cleaved off by signal peptidase 1, giving a mature protein of 163 residues[1–3]. The only post-translational modification of MPB70 appears to be a disulfide bond linking residues C38 and C172[2]. These residue numbers, and all others in this file, correspond to the immature (pre-cleavage) form of the protein being described.

At 220 residues, MPB83 is slightly larger than MPB70, and contains a 23-residue N-terminal signal peptide that is cleaved by signal peptidase II[3,4]. Beginning at position 22, the protein contains the post-translational lipidation motif LAGC, with the cysteine residue being lipidated[5]. MPB83 is also post-translationally glycosylated, with O-mannose linkages at two adjacent threonine residues (T48 and T49)[6].

Previously, it was observed that *mpb70* and *mpb83* are paralogues with significant sequence identity[7]. When a Needleman-Wunsch global alignment[8] between their coding sequences was performed using the EMBOSS[9] program `needle`, 63.6% of the alignment positions were identical, while 18.0% of the positions were gaps and 18.4% were mismatches (Figure 1). Most of the gaps occurred in *mpb70* near the beginning of the alignment. In a global alignment between the corresponding proteins, 60.5% of the alignment positions were matches, 9.4% were conservative substitutions, 14.8% were non-conservative substitutions, and 14.8% were gaps (Figure 2).

2 Structures of MPB70 and MPB83

The three-dimensional structure of MPB70 has been determined using nuclear magnetic resonance spectroscopy[2]. It contains a single β-barrel structure consisting of 7 antiparallel β-strands (Figure 3A). Half of the β-barrel is exposed to the solvent, while the other half forms part of the hydrophobic core of the protein. Residues in the β-barrel that are most solvent-accessible include 144–147, 153–158, 163–165, and 168–173.

```
mpb70    ATG---AAGGTAAAG---------------------------------AACACAATTGCG    24
mpb83    ATGATCAACGTTCAGGCCAAACCGGCCGCAGCAGCGAGCCTCGCAGCCATCGCGATTGCG    60

mpb70    -------------------GC----AACCAGTTTCG-------------------CGGCG    42
mpb83    TTCTTAGCGGGTTGTTCGAGCACCAAACCCGTGTCGCAAGACACCAGCCCGAAACCGGCG    120

mpb70    GCCGGCCTGGCGGCTC---------TGGCGG---TGGCTGTCTCACCGCCGGCGGCCGCA    90
mpb83    ACCAGCCCGGCGGCGCCCGTTACCACGGCGGCAATGGCTG----ACC--------CCGCA    168

mpb70    GGCGATCTGGTGGGCCCGGGCTGCGCGGAATACGCGGCAGC-CAATCCCACTGGGCCGGC    149
mpb83    GCGGACCTGATTGGTCGTGGGTGCGCGCAATACGCGGC-GCAAAATCCCACCGGTCCCGG    227

mpb70    CTCGGTGCAGGGAATGTCGCAGGACCCGGTCGCGGTGGCGGCCTCGAACAATCCGGAGTT    209
mpb83    ATCGGTGGCCGGAATGGCGCAAGACCCGGTCGCTACCGCGGCTTCCAACAACCCGATGCT    287

mpb70    GACAACGCTGACGGCTGCACTGTCGGGCCAGCTCAATCCGCAAGTAAACCTGGTGGACAC    269
mpb83    CAGTACCCTGACCTCGGCTCTGTCGGGCAAGCTGAACCCGGATGTGAATCTGGTCGACAC    347

mpb70    CCTCAACAGCGGTCAGTACACGGTGTTCGCACCGACCAACGCGGCATTTAGCAAGCTGCC    329
mpb83    CCTCAACGGCGGCGAGTACACCGTTTTCGCCCCCACCAACGCCGCATTCGACAAGCTGCC    407

mpb70    GGCATCCACGATCGACGAGCTCAAGACCAATTCGTCA--CTGCTGACCAGCATCCTGACC    387
mpb83    GGCGGCCACTATCGATCAACTCAAGACTGA--CGCCAAGCTGCTCAGCAGCATCCTGACC    465

mpb70    TACCACGTAGTGGCCGGCCAAACCAGCCCGGCCAACGTCGTCGGCACCCGTCAGACCCTC    447
mpb83    TACCACGTGATAGCCGGCCAGGCGAGTCCGAGCAGGATCGACGGCACCCATCAGACCCTG    525

mpb70    CAGGGCGCCAGCGTGACGGTGACCGGTCAGG-----GTAACAGCCTCAAGGTCGGTAACG    502
mpb83    CAAGGTGCCGACCTGACGGTGA-----TAGGCGCCCGCGACGACCTCATGGTCAACAACG    580

mpb70    CCGACGTCGTCTGTGGTGGGGTGTC-TACCGCCAACGCGACGGTGTACATGATTGACAGC    561
mpb83    CCGGTTTGGTATGTGGCGGAGT-TCACACCGCCAACGCGACGGTGTACATGATCGATACG    639

mpb70    GTGCTAATGCCTCCGGC---GTAA    582
mpb83    GTGCTGATGCCCCCGGCACAGTAA    663
```

Figure 1: Pairwise global alignment between the *mpb70* and *mpb83* coding sequences from *M. bovis* strain AF2122/97. Alignment positions that contain a match are coloured black, while those that contain a mismatch or gap are coloured red. The number at the end of each row indicates the position in the original sequence of the last base in that row. The global alignment was produced using the EMBOSS[9] program `needle`, while the visual representation was produced using the EMBOSS program `prettyplot`.

MPB70 also contains eight α-helices, which are largely packed against the solvent-inaccessible portion of the β-barrel (Figure 3A).

The structure of MPB83 has not yet been experimentally determined. Thus, the homology-modeling software SWISS-MODEL[10] was used to predict the structure of MPB83 based on its homology to MPB70. Only the portions of MPB70 and MPB83 that are homologous to one another were included in the modeling process; this included residues 32–193 of MPB70 and residues 58-220 of MPB83 (see Figure 2). The model built by SWISS-MODEL was nearly identical to the experimentally-determined model of MPB70, with eight α-helices and seven antiparallel β-strands forming a β-barrel (Figure 3B).

```
MPB70    M K V K N T I A A T S F A A A G L A A L A V A - - - - - - - - - - - - - - V S P - - - - - P A A - - - - - - - - - - A G    31
MPB83    M - - - I N V Q A K P A A A A S L A A I A I A F L A G C S S T K P V S Q D T S P K P A T S P A A P V T T A A M A D P A A    57

MPB70    D L V G P G C A E Y A A A N P T G P A S V Q G M S Q D P V A V A A S N N P E L T T L T A A L S G Q L N P Q V N L V D T L    91
MPB83    D L I G R G C A Q Y A A Q N P T G P G S V A G M A Q D P V A T A A S N N P M L S T L T S A L S G K L N P D V N L V D T L    117

MPB70    N S G Q Y T V F A P T N A A F S K L P A S T I D E L K T N S S L L T S I L T Y H V V A G Q T S P A N V V G T R Q T L Q G    151
MPB83    N G G E Y T V F A P T N A A F D K L P A A T I D Q L K T D A K L L S S I L T Y H V I A G Q A S P S R I D G T H Q T L Q G    177

MPB70    A S V T V T G Q G N S L K V G N A D V V C G G V S T A N A T V Y M I D S V L M P P A -    193
MPB83    A D L T V I G A R D D L M V N N A G L V C G G V H T A N A T V Y M I D T V L M P P A Q    220
```

Figure 2: Pairwise global alignment between the sequences of the MPB70 and MPB83 proteins from *M. bovis* strain AF2122/97. For further details, see the caption describing Figure 1.

3 Functions of MPB70 and MPB83

While MPB70 and MPB83 have been extensively characterized as antigens, much less is known about their functions. MPB70 is secreted[11], while MPB83 is anchored to the cell surface[12]. Both exhibit sequence homology to the FAS1 domain, which is a component of several proteins (such as fasciclin I) involved in cell adhesion[13]. It has been hypothesized that MPB70 and MPB83 may contribute to osteitis after tuberculosis infection or vaccination by interacting with periostin[3,14], which is a protein that contains multiple FAS1 domains, is found on the surface of osteoblasts, and is thought to be involved in bone formation and repair[15]. Currently, however, there appears to be no direct evidence to support this hypothesis. Beyond this, little is known about the function of these proteins, with no function-related keywords, gene ontology terms[16], or other functional annotations being associated with either protein[17]. A search for potentially more well-characterized bacterial homologues of MPB70 and MPB83 was performed using four iterations of position-specific iterative `BLAST` (`PSI-BLAST`)[18]; however, little additional information was obtained, with most hits being proteins of unknown function (e.g., "hypothetical protein") or proteins annotated as "fasciclin" or "cell surface protein".

4 Homologues of MPB70 and MPB83 in other bacteria

Homologues of MPB70 and MPB83 are present in a wide variety of bacterial lineages. According to the Microbial Genome Database[19,20], in addition to actinobacteria (the phylum to which *M. bovis* belongs), homologues of MPB70 and MPB83 are found in bacteria from several other phyla, including bacteroidetes, cyanobacteria, firmicutes, planctomycetes, and proteobacteria. It is not clear whether these homologues are orthologues, or whether they have functions different from those of MPB70 and MPB83. Despite the presence of homologues in several lineages, MPB70 and MPB83 are absent from some species that are closely related to *M. bovis*, such as *Mycobacterium avium*[21].

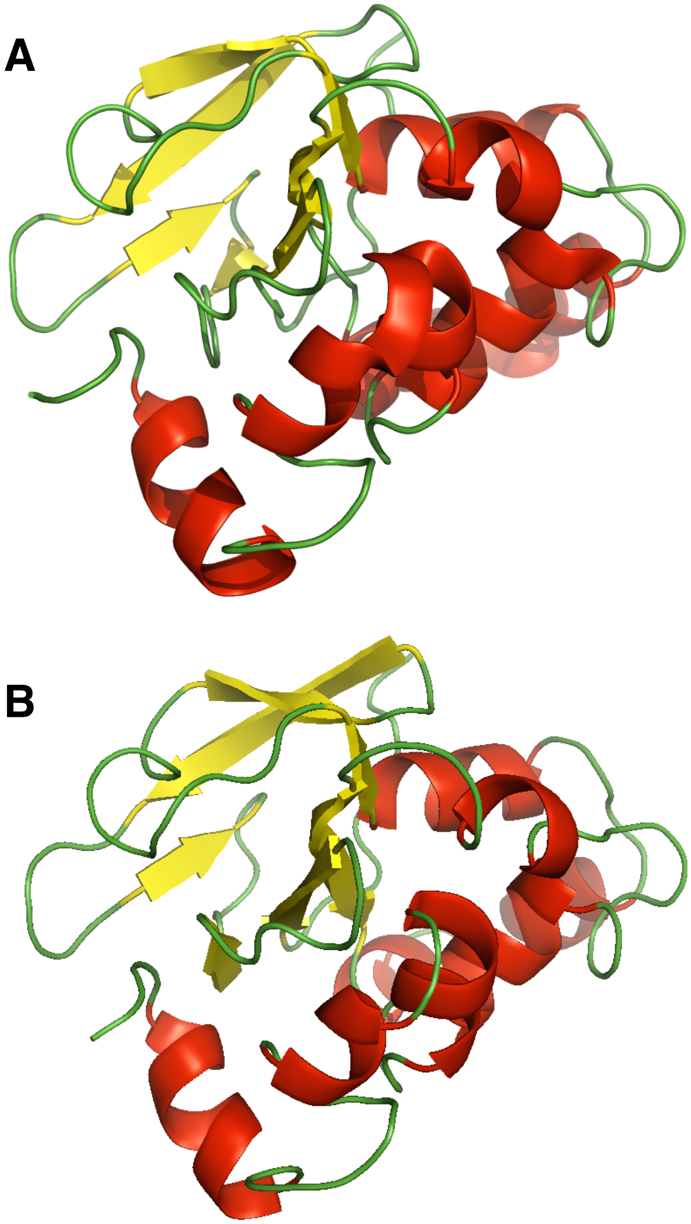

Figure 3: Cartoon representations of the three-dimensional structures of MPB70 and MPB83 after cleavage of their signal peptides. α-helices are represented by red ribbons, β-strands are represented by yellow arrows, and loops are represented by narrow green cylinders. (A) Structure of MPB70. The image was generated using PyMol[22] from the Protein Data Bank[23] entry 1NYO[2]. (B) Predicted structure of MPB83. The image was generated from a structure predicted by SWISS-MODEL[10] using the structure of MPB70 as a template.

5 Regulators of mpb70 and mpb83 expression

Two genes, *sigK* and *rskA*, encode proteins—sigma factor K (SigK) and regulator of sigma factor K (RskA), respectively—that have been found to control the expression of *mpb70* and *mpb83*. In a comparison of several different *M. bovis* strains, some of which expressed *mpb70* and *mpb83* in high amounts and some in low amounts, Charlet et al.[24] found that strains in the former group expressed *sigK* in high amounts, while strains in the latter group contained a mutation in the third position of the start codon of *sigK* that caused its expression to be very low. When low-producing strains were complemented with a non-mutated *sigK* gene, expression of *mpb70* and *mpb83* was similar to those of the high-expressing strains[24]. Thus, SigK appears to be a positive regulator of *mpb70* and *mpb83* expression. As a transcription factor, SigK contains both a DNA-binding domain and an RNA polymerase-binding domain[25]. DNA binding sites for SigK were found to be located at promoter boxes approximately 10 residues and 35 residues upstream of the transcription start site for both *mpb70* and *mpb83*[26].

Another regulator of *mpb70* and *mpb83*, RskA, has been investigated in the context of the differential expression of these genes in *M. bovis* strain AF2122/97 compared to *Mycobacterium tuberculosis* strain H37Rv. (In *M. tuberculosis*, *mpb70* and *mpb83* are conventionally called *mpt70* and *mpt83*, respectively; however, for simplicity, they will be referred to here by their *M. bovis*-specific names). Specifically, it was observed that *M. bovis* strain AF2122/97 expresses high amounts of *mpb70* and *mpb83*, while the expression of these genes in *M. tuberculosis* strain H37Rv is low, despite their *sigK* sequences being identical[27]. The authors hypothesized that *rskA*, a gene located physically close to *sigK* in the genome, was responsible for this difference. It was found that RskA in *M. bovis* contained two amino acid substitutions (G107D and G184E) relative to its counterpart in *M. tuberculosis*. When *M. bovis* was complemented with the version of *rskA* found in *M. tuberculosis*, expression of *mpb70* and *mpb83* was greatly reduced. To provide further evidence that RskA represses the transcription of *mpb70* and *mpb83*, the authors deleted the *rskA* gene in *M. tuberculosis*, which caused it to express *mpb70* and *mpb83* in quantities similar to *M. bovis* strain AF2122/97[27].

RskA represses the transcription of *mpb70* and *mpb83* by forming a complex with SigK that blocks both its DNA-binding domain and its RNA polymerase-binding domain, rendering it inactive[25]. Under oxidizing conditions, the SigK-RskA complex is stabilized by a disulfide bond between residues C133 and C183 of SigK; under reducing conditions, however, this disulfide bond is broken, causing SigK and RskA to dissociate[25]. SigK has thus been described as a redox sensor[25].

The structures of SigK and the cytoplasmic domain of RskA have been determined in complex with one another using X-ray crystallography[25] (Figure 4). SigK contains two domains, each with four α-helices. The cytoplasmic portion of RskA contains four α-helices. Three of these are sandwiched by the two domains of SigK, while the fourth lies on the outside of the complex near the N-terminus of SigK.

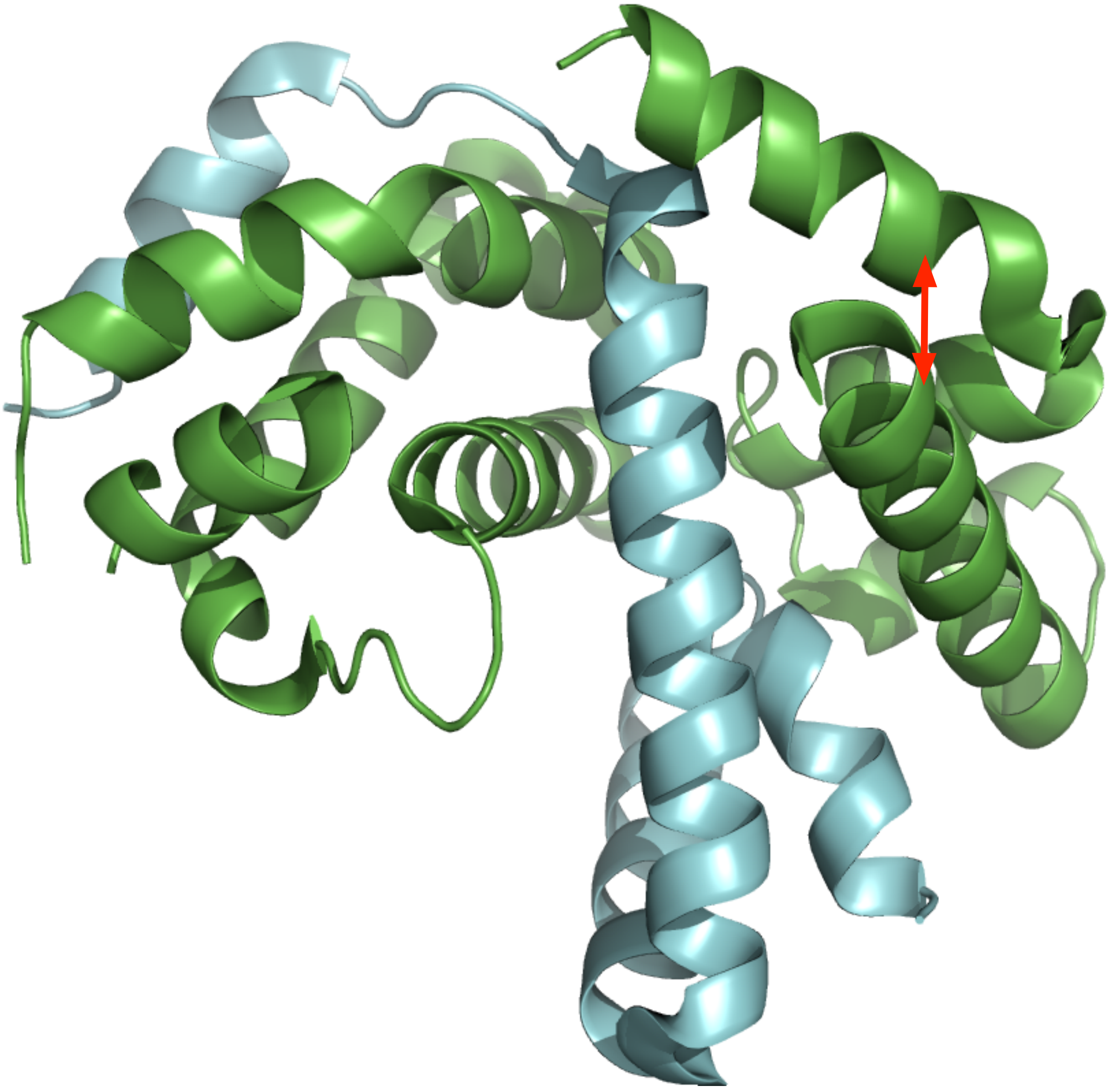

Figure 4: Cartoon representation of the three-dimensional structure of the SigK-RskA complex. SigK is shown in green, while RskA is shown in blue. The red arrow represents the approximate location of the disulfide bond in SigK. The image was generated using PyMol[22] from the Protein Data Bank[23] entry 4NQW[25].

References

1. Terasaka, K. *et al.* Complete nucleotide sequence of immunogenic protein MPB70 from *Mycobacterium bovis* BCG. *FEMS Microbiol Lett* **49,** 273–6 (1989).
2. Carr, M. D. *et al.* Solution structure of the *Mycobacterium tuberculosis* complex protein MPB70: from tuberculosis pathogenesis to inherited human corneal disease. *J Biol Chem* **278,** 43736–43 (2003).
3. Wiker, H. G. MPB70 and MPB83—major antigens of *Mycobacterium bovis*. *Scand J Immunol* **69,** 492–9 (2009).
4. Petersen, T. N., Brunak, S., von Heijne, G. & Nielsen, H. SignalP 4.0: discriminating signal peptides from transmembrane regions. *Nat Methods* **8,** 785–6 (2011).
5. Vosloo, W. *et al.* Characterisation of a lipoprotein in *Mycobacterium bovis* (BCG) with sequence similarity to the secreted protein MPB70. *Gene* **188,** 123–8 (1997).
6. Michell, S. L. *et al.* The MPB83 antigen from *Mycobacterium bovis* contains O-linked mannose and (1->3)-mannobiose moieties. *J Biol Chem* **278,** 16423–32 (2003).
7. Harboe, M., Nagai, S., Wiker, H. G., Sletten, K. & Haga, S. Homology between the MPB70 and MPB83 proteins of *Mycobacterium bovis* BCG. *Scand J Immunol* **42,** 46–51 (1995).
8. Needleman, S. B. & Wunsch, C. D. A general method applicable to the search for similarities in the amino acid sequence of two proteins. *J Mol Biol* **48,** 443–53 (1970).
9. Rice, P., Longden, I. & Bleasby, A. EMBOSS: the European Molecular Biology Open Software Suite. *Trends Genet* **16,** 276–7 (2000).

10. Biasini, M. *et al.* SWISS-MODEL: modelling protein tertiary and quaternary structure using evolutionary information. *Nucleic Acids Res* **42,** W252–8 (2014).

11. Nagai, S., Matsumoto, J. & Nagasuga, T. Specific skin-reactive protein from culture filtrate of *Mycobacterium bovis* BCG. *Infect Immun* **31,** 1152–60 (1981).

12. Harboe, M. *et al.* MPB70 and MPB83 as indicators of protein localization in mycobacterial cells. *Infect Immun* **66,** 289–96 (1998).

13. Clout, N. J., Tisi, D. & Hohenester, E. Novel fold revealed by the structure of a FAS1 domain pair from the insect cell adhesion molecule fasciclin I. *Structure* **11,** 197–203 (2003).

14. Ulstrup, J. C., Jeansson, S., Wiker, H. G. & Harboe, M. Relationship of secretion pattern and MPB70 homology with osteoblast-specific factor 2 to osteitis following *Mycobacterium bovis* BCG vaccination. *Infect Immun* **63,** 672–5 (1995).

15. Merle, B. & Garnero, P. The multiple facets of periostin in bone metabolism. *Osteoporos Int* **23,** 1199–212 (2012).

16. Ashburner, M. *et al.* Gene ontology: tool for the unification of biology. *Nat Genet* **25,** 25–9 (2000).

17. UniProt Consortium. The Universal Protein Resource (UniProt). *Nucleic Acids Res* **36,** D190–5 (2008).

18. Altschul, S. F. *et al.* Gapped BLAST and PSI-BLAST: a new generation of protein database search programs. *Nucleic Acids Res* **25,** 3389–402 (1997).

19. Uchiyama, I. MBGD: microbial genome database for comparative analysis. *Nucleic Acids Res* **31,** 58–62 (2003).

20. Uchiyama, I. MBGD: a platform for microbial comparative genomics based on the automated construction of orthologous groups. *Nucleic Acids Res* **35,** D343–6 (2007).
21. Waters, W. R. *et al.* Development and evaluation of an enzyme-linked immunosorbent assay for use in the detection of bovine tuberculosis in cattle. *Clin Vaccine Immunol* **18,** 1882–8 (2011).
22. Schrödinger, LLC. The PyMOL Molecular Graphics System, Version 1.3r1. (2010).
23. Berman, H. M. *et al.* The Protein Data Bank. *Nucleic Acids Res* **28,** 235–42 (2000).
24. Charlet, D. *et al.* Reduced expression of antigenic proteins MPB70 and MPB83 in *Mycobacterium bovis* BCG strains due to a start codon mutation in *sigK*. *Mol Microbiol* **56,** 1302–13 (2005).
25. Shukla, J., Gupta, R., Thakur, K. G., Gokhale, R. & Gopal, B. Structural basis for the redox sensitivity of the *Mycobacterium tuberculosis* SigK-RskA σ-anti-σ complex. *Acta Crystallogr Biol Crystallogr* **70,** 1026–36 (2014).
26. Rodrigue, S. *et al.* Identification of mycobacterial sigma factor binding sites by chromatin immunoprecipitation assays. *J Bacteriol* **189,** 1505–13 (2007).
27. Saïd-Salim, B., Mostowy, S., Kristof, A. S. & Behr, M. A. Mutations in *Mycobacterium tuberculosis* Rv0444c, the gene encoding anti-SigK, explain high level expression of MPB70 and MPB83 in *Mycobacterium bovis*. *Mol Microbiol* **62,** 1251–63 (2006).

Supplementary Discussion S2: Mutations in *mpb70*, *mpb83*, *sigK*, and *rskA* that could cause false negatives by the IDEXX ELISA

Several types of mutations in *mpb70*, *mpb83*, *sigK*, and *rskA* could explain false negatives by the IDEXX ELISA. These mutations can be divided into two categories: those that could prevent the anti-MPB70 or anti-MPB83 antibodies produced by the bovine host from interacting with the versions of those proteins used in the IDEXX ELISA, and those that could prevent the bovine host from generating anti-MPB70 or anti-MPB83 antibodies altogether. Many of these potential mutations relate to specific sequence- or structure-related attributes of MPB70, MPB83, SigK, and RskA (see Supplementary Discussion S1 online).

Potential mutations falling into the first category are as follows. For ease of discussion, some potential mutations are described in terms of the genes themselves, whereas others are described with reference to changes in their protein products.

- **Substitutions in MPB70 or MPB83 epitopes.** These could cause the antibodies generated by the infected bovine to be reactive to the mutated versions of the proteins, but not to the versions used in the IDEXX ELISA. Although it is not clear which regions of MPB70 and MPB83 are typically recognized by antibodies, solvent-accessible regions (such as residues 114–117, 123–128, 133–135, and 138–143 in MPB70; see Supplementary Discussion S1 online) would be more likely to be epitopes.
- **Substitutions in the glycosylated residues of MPB83 (T48 and T49).** Such mutations could prevent MPB83 from being glycosylated, and it is possible that antibodies that recognize the unglycosylated form of the protein would not recognize the glycosylated form in the IDEXX ELISA. Substitutions in residues near the glycosylation sites, which may act as a recognition motif for the enzyme catalyzing the glycosylation reactions, could have the same effect.
- **Frameshift mutations in the *mpb70* or *mpb83* coding sequences.** An insertion or deletion that changes the reading frame would result in the production of a very different protein product. Antibodies that recognize this product would be unlikely to recognize the corresponding protein used in the IDEXX ELISA.

Potential mutations falling into the second category are as follows.

- **Mutations that alter the signal peptide of MPB70 or MPB83.** These could prevent them from being secreted (for MPB70) or anchored to the cell surface (for MPB83), making them inaccessible to the host's antibodies.
- **Missense mutations in the start codon of *mpb70* or *mpb83*.** These would prevent their transcription, and thus no corresponding protein would be produced.
- **Substitutions in the lipidation motif (residues 22–25) of MPB83.** These could prevent the lipidation of MPB83, preventing it from becoming anchored to the cell surface.

- **Missense mutations in the start codon of *sigK*.** These would prevent its transcription, and without SigK, *mpb70* and *mpb83* would no longer be transcribed.
- **Missense mutations, insertions, deletions, or nonsense mutations in the *sigK* coding sequence.** Such mutations could render its protein product nonfunctional, preventing *mpb70* or *mpb83* from being transcribed.
- **The mutations D107G and E184G in RskA.** As described in Supplementary Discussion S1 online, it has been shown that the presence of Asp and Glu in positions of 107 and 184, respectively, of RskA renders it non-functional, whereas it is functional when glycine residues are present in both positions. As RskA negatively regulates the expression of *mpb70* and *mpb83*, the mutations D107G and E184G could cause only small amounts of MPB70 and MPB83 to be produced.
- **Nonsense mutations in the *mpb70* or *mpb83* coding sequences.** A premature stop codon would result in a shortened protein product, and antibodies may not be elicited to the truncated protein.
- **Mutations in the regulatory regions upstream of the *mpb70* or *mpb83* coding sequences.** Mutations in these regions could reduce the ability of SigK to bind, preventing the transcription of these genes.

Supplementary Table S4: Sequence variation in the extended *mpb70* coding sequences

Strain	-244	-225	-210	-197	-173	-54	47	48	50	57	396	528	+25	+187	+353	+402	+403	+408	+412	+413	+416	+438
AF2122/97 (UK)	C	G	G	C	G	G	G	C	T	T	A	T	C	C	G	G	C	C	C	C	A	C
AF-16-01370-12 (UK)	.	.	.	G	.	.	.	.	.	.	.	.	.	.	.	.	.	.	.	.	.	.
AFT-16-02181-13 (UK)	.	.	.	.	.	.	.	.	.	.	.	.	.	.	.	.	.	.	.	.	.	T
01-2509 (USA)	.	.	.	.	.	.	.	.	.	.	.	.	.	.	.	.	.	.	A	G	.	.
07-6182 (USA)	G	.	.	.	.	A	.	.	.	.	.	.	A	.	.	.	.	.	.	.	.	.
12-9603 (USA)	.	.	.	.	.	.	A	.	.	.	.	.	.	.	.	.	.	A	.	.	.	.
02-2201 (USA)	.	.	.	.	.	A	.	.	.	.	.	.	.	.	.	.	.	.	.	.	.	.
02-6259 (USA)	.	.	.	.	.	A	.	.	.	.	.	.	.	.	.	.	.	.	.	.	.	.
03-0248 (USA)	.	.	.	.	.	A	.	.	.	.	.	.	.	.	.	.	.	.	.	.	.	.
03-1852 (USA)	.	.	.	.	.	A	.	.	.	.	.	.	.	.	.	.	.	.	.	.	.	.
03-2614 (USA)	.	.	.	.	.	A	.	.	.	.	.	.	.	.	.	.	.	.	.	.	.	.
03-2617 (USA)	.	.	.	.	.	A	.	.	.	.	.	.	.	.	.	.	.	.	.	.	.	.
03-2618 (USA)	.	.	.	.	.	A	.	.	.	.	.	.	.	.	.	.	.	.	.	.	.	.
03-2620 (USA)	.	.	.	.	.	A	.	.	.	.	.	.	.	.	.	.	.	.	.	.	.	.
03-2622 (USA)	.	.	.	.	.	A	.	.	.	.	.	.	.	.	.	.	.	.	.	.	.	.
03-2623 (USA)	.	.	.	.	.	A	.	.	.	.	.	.	.	.	.	.	.	.	.	.	.	.
03-2627 (USA)	.	.	.	.	.	A	.	.	.	.	.	.	.	.	.	.	.	.	.	.	.	.
03-2628 (USA)	.	.	.	.	.	A	.	.	.	.	.	.	.	.	.	.	.	.	.	.	.	.
03-2629 (USA)	.	.	.	.	.	A	.	.	.	.	.	.	.	.	.	.	.	.	.	.	.	.
03-2994 (USA)	.	.	.	.	.	A	.	.	.	.	.	.	.	.	.	.	.	.	.	.	.	.
03-2995 (USA)	.	.	.	.	.	A	.	.	.	.	.	.	.	.	.	.	.	.	.	.	.	.
03-4006 (USA)	.	.	.	.	.	A	.	.	.	.	.	.	.	.	.	.	.	.	.	.	.	.
06-2501 (USA)	.	.	.	.	.	A	.	.	.	.	.	.	.	.	.	.	.	.	.	.	.	.
08-1587 (USA)	.	.	.	.	.	A	.	.	.	.	.	.	.	.	.	.	.	.	.	.	.	.
08-2416 (USA)	.	.	.	.	.	A	.	.	.	.	.	.	.	.	.	.	.	.	.	.	.	.
08-2429 (USA)	.	.	.	.	.	A	.	.	.	.	.	.	.	.	.	.	.	.	.	.	.	.
08-2431 (USA)	.	.	.	.	.	A	.	.	.	.	.	.	.	.	.	.	.	.	.	.	.	.
08-2434 (USA)	.	.	.	.	.	A	.	.	.	.	.	.	.	.	.	.	.	.	.	.	.	.
08-3760 (USA)	.	.	.	.	.	A	.	.	.	.	.	.	.	.	.	.	.	.	.	.	.	.
08-5176 (USA)	.	.	.	.	.	A	.	.	.	.	.	.	.	.	.	.	.	.	.	.	.	.
09-0109 (USA)	.	.	.	.	.	A	.	.	.	.	.	.	.	.	.	.	.	.	.	.	.	.
09-3201 (USA)	.	.	.	.	.	A	.	.	.	.	.	.	.	.	.	.	.	.	.	.	.	.

10-2172 (USA)	.	.	.	.	.	A	.	.	.	.	.	.	.	.	.	.	.	.	.	.	.	.
10-2603 (USA)	.	.	.	.	.	A	.	.	.	.	.	.	.	.	.	.	.	.	.	.	.	.
10-3273 (USA)	.	.	.	.	.	A	.	.	.	.	.	.	.	.	.	.	.	.	.	.	.	.
10-4566 (USA)	.	.	.	.	.	A	.	.	.	.	.	.	.	.	.	.	.	.	.	.	.	.
10-6148 (USA)	.	.	.	.	.	A	.	.	.	.	.	.	.	.	.	.	.	.	.	.	.	.
10-7034 (USA)	.	.	.	.	.	A	.	.	.	.	.	.	.	.	.	.	.	.	.	.	.	.
11-3871 (USA)	.	.	.	.	.	A	.	.	.	.	.	.	.	.	.	.	.	.	.	.	.	.
11-3877 (USA)	.	.	.	.	.	A	.	.	.	.	.	.	.	.	.	.	.	.	.	.	.	.
11-5424 (USA)	.	.	.	.	.	A	.	.	.	.	.	.	.	.	.	.	.	.	.	.	.	.
11-5428 (USA)	.	.	.	.	.	A	.	.	.	.	.	.	.	.	.	.	.	.	.	.	.	.
11-5500 (USA)	.	.	.	.	.	A	.	.	.	.	.	.	.	.	.	.	.	.	.	.	.	.
11-5501 (USA)	.	.	.	.	.	A	.	.	.	.	.	.	.	.	.	.	.	.	.	.	.	.
96-3239 (USA)	.	.	.	.	.	A	.	.	.	.	.	.	.	.	.	.	.	.	.	.	.	.
00-5480 (USA)	.	.	.	.	.	.	.	.	.	.	.	.	.	T	.	.	.	.	.	.	.	.
01-3872 (USA)	.	.	.	.	.	.	.	.	.	.	.	.	.	.	.	.	.	.	.	.	C	.
02-4544 (USA)	.	.	.	.	.	.	.	.	.	.	.	.	.	.	.	.	.	.	.	.	C	.
01-4535 (USA)	.	.	.	.	T	.	.	.	.	.	.	.	.	.	.	.	.	.	.	.	.	.
02-2284 (USA)	.	.	.	.	.	.	.	.	.	.	G	.	.	.	.	.	.	.	.	.	.	.
02-4556 (USA)	.	.	.	.	.	.	.	.	.	.	G	.	.	.	.	.	.	.	.	.	.	.
03-0196 (USA)	.	.	.	.	.	.	.	.	.	.	G	.	.	.	.	.	.	.	.	.	.	.
08-2630 (USA)	.	C	.	.	.	.	.	.	.	.	.	.	.	.	.	.	.	.	.	.	.	.
12-3930 (USA)	.	.	.	.	.	.	.	.	.	.	.	.	.	.	.	.	A	.	.	.	.	.
12-5092 (USA)	.	.	.	.	.	.	.	.	.	.	.	C	.	.	.	.	.	.	.	.	.	.
12-5103 (USA)	.	.	.	.	.	.	.	.	.	C	.	.	.	.	.	.	.	.	.	.	.	.
94-1195 (USA)	.	.	.	.	.	.	.	.	.	.	.	.	.	.	A	.	.	.	.	.	.	.
11-4613 (USA)	.	.	.	.	.	.	.	.	.	.	.	.	.	.	.	-	.	.	.	.	.	.
07-10613 (MEX)	.	.	A	.	.	A	.	.	.	.	.	.	.	.	.	.	.	.	.	.	.	.
01-2656 (MEX)	.	.	.	.	.	A	.	.	.	.	.	.	.	.	.	.	.	.	.	.	.	.
01-4280 (MEX)	.	.	.	.	.	A	.	.	.	.	.	.	.	.	.	.	.	.	.	.	.	.
02-2159 (MEX)	.	.	.	.	.	A	.	.	.	.	.	.	.	.	.	.	.	.	.	.	.	.
02-3019 (MEX)	.	.	.	.	.	A	.	.	.	.	.	.	.	.	.	.	.	.	.	.	.	.
02-3307 (MEX)	.	.	.	.	.	A	.	.	.	.	.	.	.	.	.	.	.	.	.	.	.	.
02-3401 (MEX)	.	.	.	.	.	A	.	.	.	.	.	.	.	.	.	.	.	.	.	.	.	.
02-5899 (MEX)	.	.	.	.	.	A	.	.	.	.	.	.	.	.	.	.	.	.	.	.	.	.
04-2550 (MEX)	.	.	.	.	.	A	.	.	.	.	.	.	.	.	.	.	.	.	.	.	.	.
05-6456 (MEX)	.	.	.	.	.	A	.	.	.	.	.	.	.	.	.	.	.	.	.	.	.	.
05-6593 (MEX)	.	.	.	.	.	A	.	.	.	.	.	.	.	.	.	.	.	.	.	.	.	.
05-8448 (MEX)	.	.	.	.	.	A	.	.	.	.	.	.	.	.	.	.	.	.	.	.	.	.
06-3192 (MEX)	.	.	.	.	.	A	.	.	.	.	.	.	.	.	.	.	.	.	.	.	.	.

07-7377 (MEX)	.	.	.	.	.	A	.	.	.	.	.	.	.	.	.	.	.	.	.	.	.	.
08-2906 (MEX)	.	.	.	.	.	A	.	.	.	.	.	.	.	.	.	.	.	.	.	.	.	.
08-6489 (MEX)	.	.	.	.	.	A	.	.	.	.	.	.	.	.	.	.	.	.	.	.	.	.
10-1862 (MEX)	.	.	.	.	.	A	.	.	.	.	.	.	.	.	.	.	.	.	.	.	.	.
08-4517 (MEX)	.	.	.	.	.	.	.	G	.	.	.	.	.	.	.	.	.	.	.	.	.	.
12-9259 (MEX)	.	.	.	.	.	.	.	.	A	.	.	.	.	.	.	.	.	.	.	.	.	.

Supplementary Table S5: Sequence variation in the extended *mpb83* coding sequences

Strain	-344	-338	-334	-188	36	111	585	+160	+193	+446
AF2122/97 (UK)	G	C	C	C	G	G	T	G	T	C
61-0546-02 (UK)	C	A	G	.	.	.	.	.	.	.
AF-21-06118-12 (UK)	.	.	.	.	.	.	.	.	G	.
AFT-16-01365-13 (UK)	.	.	.	.	.	.	.	.	G	.
61-3215-08 (UK)	.	.	.	T	.	.	.	.	.	.
96-3239 (USA)	.	.	.	.	.	.	.	.	G	G
00-5480 (USA)	.	.	.	.	.	.	.	.	G	.
01-1951 (USA)	.	.	.	.	.	.	.	.	G	.
01-1952 (USA)	.	.	.	.	.	.	.	.	G	.
01-1954 (USA)	.	.	.	.	.	.	.	.	G	.
02-2201 (USA)	.	.	.	.	.	.	.	.	G	.
02-6259 (USA)	.	.	.	.	.	.	.	.	G	.
03-0248 (USA)	.	.	.	.	.	.	.	.	G	.
03-1852 (USA)	.	.	.	.	.	.	.	.	G	.
03-2614 (USA)	.	.	.	.	.	.	.	.	G	.
03-2617 (USA)	.	.	.	.	.	.	.	.	G	.
03-2618 (USA)	.	.	.	.	.	.	.	.	G	.
03-2620 (USA)	.	.	.	.	.	.	.	.	G	.
03-2622 (USA)	.	.	.	.	.	.	.	.	G	.
03-2623 (USA)	.	.	.	.	.	.	.	.	G	.
03-2627 (USA)	.	.	.	.	.	.	.	.	G	.
03-2628 (USA)	.	.	.	.	.	.	.	.	G	.
03-2629 (USA)	.	.	.	.	.	.	.	.	G	.
03-2994 (USA)	.	.	.	.	.	.	.	.	G	.
03-2995 (USA)	.	.	.	.	.	.	.	.	G	.
03-4006 (USA)	.	.	.	.	.	.	.	.	G	.
06-2501 (USA)	.	.	.	.	.	.	.	.	G	.
07-6182 (USA)	.	.	.	.	.	.	.	.	G	.
08-1587 (USA)	.	.	.	.	.	.	.	.	G	.
08-2416 (USA)	.	.	.	.	.	.	.	.	G	.
08-2429 (USA)	.	.	.	.	.	.	.	.	G	.
08-2431 (USA)	.	.	.	.	.	.	.	.	G	.

08-2434 (USA)	.	.	.	.	.	.	.	.	G	.
08-3760 (USA)	.	.	.	.	.	.	.	.	G	.
08-5176 (USA)	.	.	.	.	.	.	.	.	G	.
10-2172 (USA)	.	.	.	.	.	.	.	.	G	.
10-2603 (USA)	.	.	.	.	.	.	.	.	G	.
10-7034 (USA)	.	.	.	.	.	.	.	.	G	.
11-3871 (USA)	.	.	.	.	.	.	.	.	G	.
11-3877 (USA)	.	.	.	.	.	.	.	.	G	.
11-5424 (USA)	.	.	.	.	.	.	.	.	G	.
11-5428 (USA)	.	.	.	.	.	.	.	.	G	.
11-5500 (USA)	.	.	.	.	.	.	.	.	G	.
11-5501 (USA)	.	.	.	.	.	.	.	.	G	.
94-1195 (USA)	.	.	.	.	.	.	.	.	G	.
02-2284 (USA)	.	.	.	.	C	.	.	.	.	.
07-11952 (USA)	.	.	.	.	.	.	.	C	.	.
11-5713 (USA)	.	.	.	.	.	.	G	.	.	.
01-2656 (MEX)	.	.	.	.	.	.	.	.	G	.
01-4280 (MEX)	.	.	.	.	.	.	.	.	G	.
02-2159 (MEX)	.	.	.	.	.	.	.	.	G	.
02-3019 (MEX)	.	.	.	.	.	.	.	.	G	.
02-3307 (MEX)	.	.	.	.	.	.	.	.	G	.
02-3401 (MEX)	.	.	.	.	.	.	.	.	G	.
02-5899 (MEX)	.	.	.	.	.	.	.	.	G	.
04-2550 (MEX)	.	.	.	.	.	.	.	.	G	.
05-6456 (MEX)	.	.	.	.	.	.	.	.	G	.
05-6593 (MEX)	.	.	.	.	.	.	.	.	G	.
05-8448 (MEX)	.	.	.	.	.	.	.	.	G	.
06-3192 (MEX)	.	.	.	.	.	.	.	.	G	.
07-10613 (MEX)	.	.	.	.	.	.	.	.	G	.
07-7377 (MEX)	.	.	.	.	.	.	.	.	G	.
08-2906 (MEX)	.	.	.	.	.	.	.	.	G	.
08-6489 (MEX)	.	.	.	.	.	.	.	.	G	.
10-1862 (MEX)	.	.	.	.	.	.	.	.	G	.
01-3302 (MEX)	C	.	.	.	.	.	.	.	.	.
02-1579 (MEX)	.	.	.	.	.	A	.	.	.	.

Supplementary Table S6: Sequence variation in the extended *sigK* coding sequences

Strain	474	546	+28	+32	+49	+162	+226	+238	+239	+244	+245
AF2122/97 (UK)	T	C	T	A	T	T	C	G	C	A	G
16-1397-11 (UK)	.	T	.	.	.	.	.	.	.	.	.
AF-61-03361-12 (UK)	.	T	.	.	.	.	.	.	.	.	.
10-2603 (USA)	.	.	.	.	.	.	.	.	.	C	C
00-5480 (USA)	.	.	.	.	.	.	T	.	.	.	.
01-2509 (USA)	.	.	.	.	C	.	.	.	.	.	.
07-6182 (USA)	.	.	.	.	C	.	.	.	.	.	.
12-5094 (USA)	.	.	.	.	C	.	.	.	.	.	.
13-0895 (USA)	.	.	.	.	C	.	.	.	.	.	.
08-1587 (USA)	G	.	.	.	.	.	.	.	.	.	.
08-2416 (USA)	G	.	.	.	.	.	.	.	.	.	.
08-2429 (USA)	G	.	.	.	.	.	.	.	.	.	.
08-2431 (USA)	G	.	.	.	.	.	.	.	.	.	.
08-2434 (USA)	G	.	.	.	.	.	.	.	.	.	.
08-5176 (USA)	G	.	.	.	.	.	.	.	.	.	.
09-0739 (USA)	.	.	.	.	.	.	.	C	.	.	.
12-5093 (USA)	.	.	A	.	.	.	.	.	.	.	.
01-1360 (MEX)	.	.	.	G	.	.	.	.	.	G	.
01-0050 (MEX)	.	.	.	.	C	.	.	.	.	.	.
01-0843 (MEX)	.	.	.	.	C	.	.	.	.	.	.
11-4782 (MEX)	.	.	.	.	.	.	.	.	T	.	.
12-9906 (MEX)	.	.	.	.	.	G	.	.	.	.	.

Supplementary Table S7: Sequence variation in the extended *rskA* coding sequences

Strain	-134	-62	-16	-12	6	119	183	195	196	201	202	580	+63	+267
AF2122/97 (UK)	T	C	T	A	T	T	C	G	C	A	G	T	G	G
16-1397-11 (UK)	.	T	.	.	.	.	.	.	.	.	.	.	.	.
AF-61-03361-12 (UK)	.	T	.	.	.	.	.	.	.	.	.	.	.	.
10-2603 (USA)	.	.	.	.	.	.	.	.	.	C	C	.	.	.
01-2509 (USA)	.	.	.	.	C	.	.	.	.	.	.	.	.	.
07-6182 (USA)	.	.	.	.	C	.	.	.	.	.	.	.	.	.
12-5094 (USA)	.	.	.	.	C	.	.	.	.	.	.	.	.	.
13-0895 (USA)	.	.	.	.	C	.	.	.	.	.	.	.	.	.
00-5480 (USA)	.	.	.	.	.	.	T	.	.	.	.	.	.	.
08-1587 (USA)	G	.	.	.	.	.	.	.	.	.	.	.	.	.
08-2416 (USA)	G	.	.	.	.	.	.	.	.	.	.	.	.	.
08-2429 (USA)	G	.	.	.	.	.	.	.	.	.	.	.	.	.
08-2431 (USA)	G	.	.	.	.	.	.	.	.	.	.	.	.	.
08-2434 (USA)	G	.	.	.	.	.	.	.	.	.	.	.	.	.
08-5176 (USA)	G	.	.	.	.	.	.	.	.	.	.	.	.	.
09-0739 (USA)	.	.	.	.	.	.	.	C	.	.	.	.	.	.
12-5092 (USA)	.	.	.	.	.	.	.	.	.	.	.	G	.	.
12-5093 (USA)	.	.	A	.	.	.	.	.	.	.	.	.	.	.
01-1360 (MEX)	.	.	.	G	.	.	.	.	.	G	.	.	.	.
02-6427 (MEX)	.	.	.	.	.	.	.	.	.	.	.	.	.	A
01-0050 (MEX)	.	.	.	.	C	.	.	.	.	.	.	.	.	A
01-0843 (MEX)	.	.	.	.	C	.	.	.	.	.	.	.	.	.
03-2704 (MEX)	.	.	.	.	.	.	.	.	.	.	.	.	A	.
11-4782	.	.	.	.	.	.	.	.	T	.	.	.	.	.

(MEX)														
12-9906<br>(MEX)	.	.	.	.	.	G	.	.	.	.	.	.	.	.

ACCCCGCGGGCCGCACTTACTCCCGAGACGTACTTCGGAGTCGGCAAGGTGGTCAACTAC
GGCGGCGGCGGCGCATATGACGAAGGGTCGGCCGTGTTTGACTACCCGCCCAGTTTGGCA
GCCAACAGCTTTGCACTGCGCGGCCGGTGGGCGCTGGACTATCAGGGTGCCACGTCCGAC
GGCAACGACGCCGCTATCAAATTGAATTACCACGCCAAAGACGTCTACATCGTTGTCGGT
GGCACCGGCACCCTCACGGTCGTGAGGGACGGAAAGCCAGCCACACTACCGATCAGCGGG
CCGCCGACCACCCATCAGGTGGTCGCCGGCGATCGGCTGGCGTCCGAAACACTTGAGGTG
CGGCCCAGCAAGGGGCTACAGGTTTTTTCCTTCACCTACGGATGAATATCCATCCAAGAC
CCGGACGGCTCCGAAGAAATCATGTCGGGGGTAGCGAGACGGCACAAGCCGCCGTCTCCG
GCAGCGAAGGAGTGAACGGCATGAAGGTAAAGAACACAATTGCGGCAACCAGTTTCGCGG
CGGCCGGCCTGGCGGCTCTGGCGGTGGCTGTCTCACCGCCGGCGGCCGCAGGCGATCTGG
TGGGCCCGGGCTGCGCGGAATACGCGGCAGCCAATCCCACTGGGCCGGCCTCGGTGCAGG
GAATGTCGCAGGACCCGGTCGCGGTGGCGGCCTCGAACAATCCGGAGTTGACAACGCTGA
CGGCTGCACTGTCGGGCCAGCTCAATCCGCAAGTAAACCTGGTGGACACCCTCAACAGCG
GTCAGTACACGGTGTTCGCACCGACCAACGCGGCATTTAGCAAGCTGCCGGCATCCACGA
TCGACGAGCTCAAGACCAATTCGTCACTGCTGACCAGCATCCTGACCTACCACGTAGTGG
CCGGCCAAACCAGCCCGGCCAACGTCGTCGGCACCCGTCAGACCCTCCAGGGCGCCAGCG
TGACGGTGACCGGTCAGGGTAACAGCCTCAAGGTCGGTAACGCCGACGTCGTCTGTGGTG
GGGTGTCTACCGCCAACGCGACGGTGTACATGATTGACAGCGTGCTAATGCCTCCGGCGT
AATCGTCCGCGGAGGCCGCCGACCCGCCCGAGAGCGACTGAGCATGTGCCAGAATGTTCG
GGCAGTGGGAGTTCGACGTCAGTCCAACCGGAGGAATCGCCGTGGCAAGTACCGAGGTGG
AGCACTTCGCCGGCTCGCAACATGAGGTCGACACCGCCGAGGTTCCATCTGCAGCGTGGG
GGCGGAGCCGGATCGATCACCGCACCTGGCACATCGTCGGCCTGTGCATCTTCGGCTTCC
TGCTGGCGATGCTGCGGGGCAACCACGTCGGCCACGTCGAGGACTGGTTCCTGATCACGT
TTGCCGCAGTCGTGCTGTTCGTCTTGGCGCGCGACTTGTGGGGCCGACGACGCGGCTGGA
TCAGATAGCCAGCACACCGTTCGGTGTGCCCGACCCGGTCAGCGCCGCACCCGCCGAAAC
CAGGTACCGGCGAAGGCACCGACCACCAGCACAACCAGCAACACCGCCCAAGGCCATGCA
CCGTGCTGGTTAACCCAGCCAG

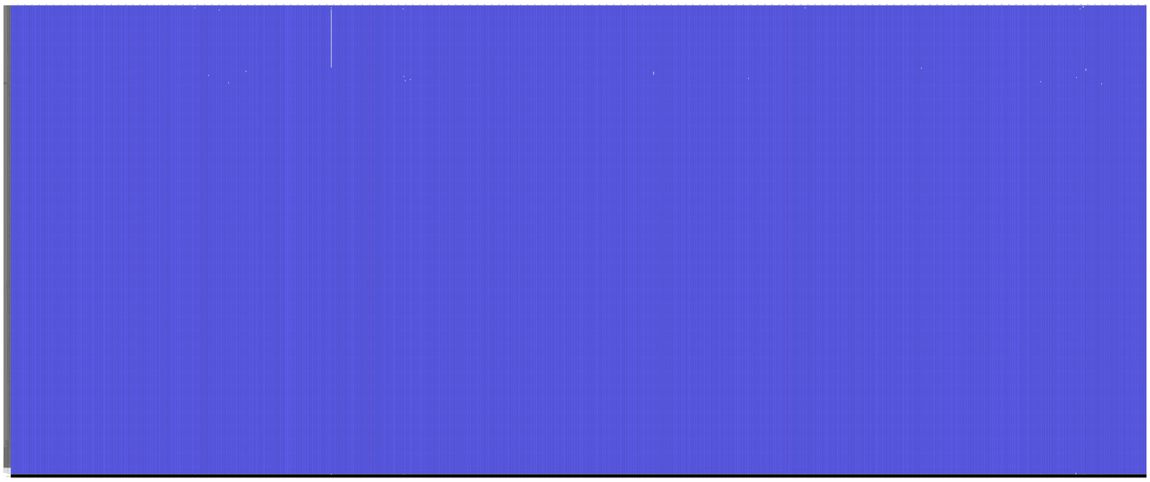

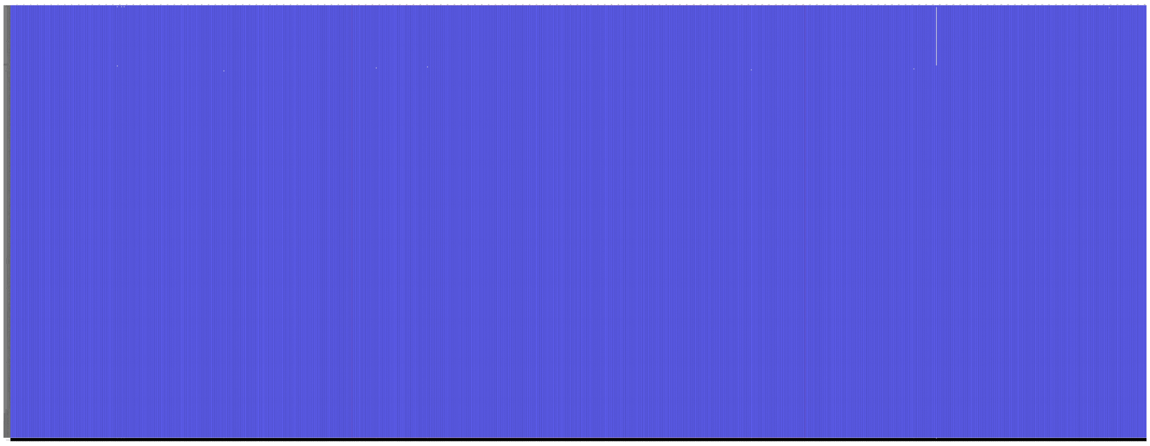

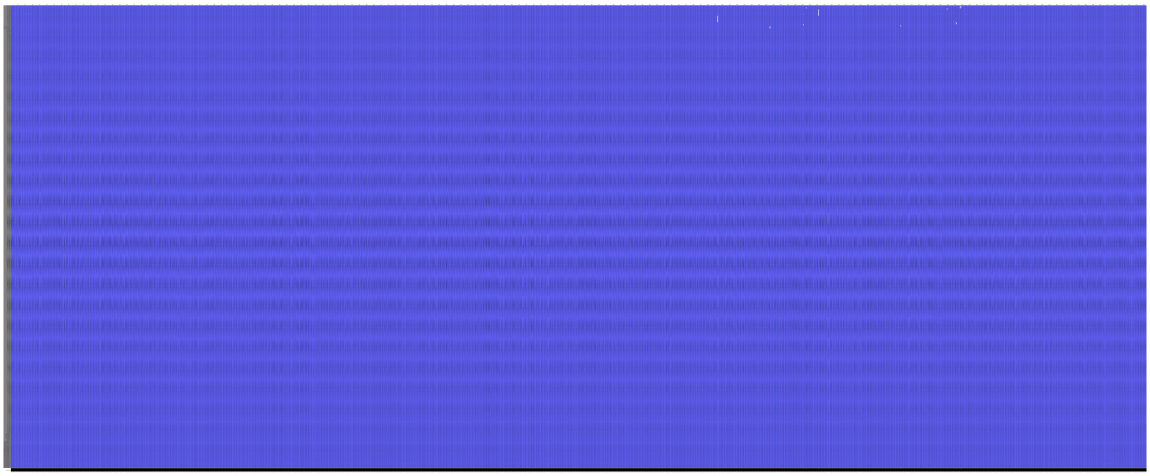

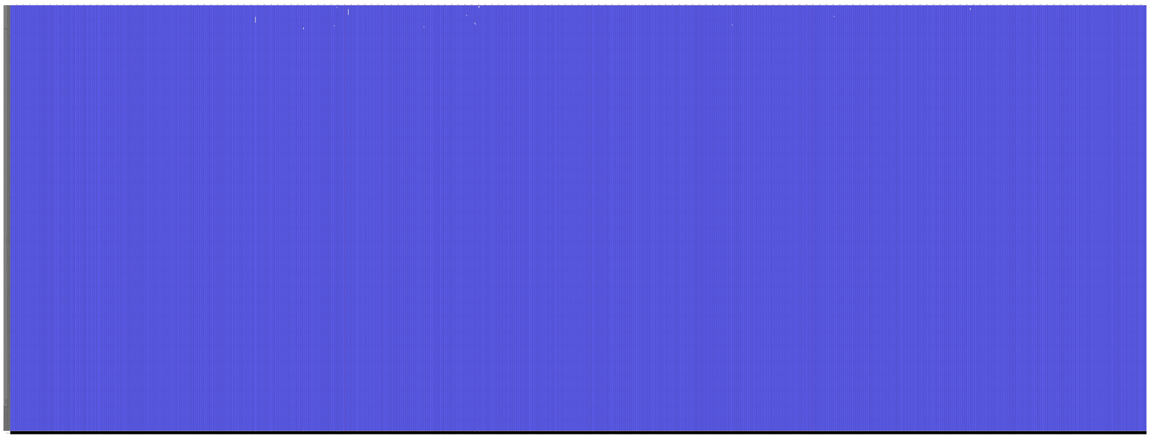

Supplement: Supplementary Information [file srep22763-s1.pdf]
